# *Osmunda pulchella* sp. nov. from the Jurassic of Sweden—reconciling molecular and fossil evidence in the phylogeny of modern royal ferns (Osmundaceae)

**DOI:** 10.1186/s12862-015-0400-7

**Published:** 2015-06-30

**Authors:** Benjamin Bomfleur, Guido W. Grimm, Stephen McLoughlin

**Affiliations:** Department of Palaeobiology, Swedish Museum of Natural History, Stockholm, Sweden; Department of Palaeontology, University of Vienna, Vienna, Austria

**Keywords:** Calcification, Evolutionary placement, Fern evolution, Organelle preservation, Osmundales, *Osmundastrum*, Outgroup, Paraphyly, Permineralization, Phylogenetic networks

## Abstract

**Background:**

The classification of royal ferns (Osmundaceae) has long remained controversial. Recent molecular phylogenies indicate that *Osmunda* is paraphyletic and needs to be separated into *Osmundastrum* and *Osmunda s.str.* Here, however, we describe an exquisitely preserved Jurassic *Osmunda* rhizome (*O. pulchella* sp. nov.) that combines diagnostic features of both *Osmundastrum* and *Osmunda*, calling molecular evidence for paraphyly into question. We assembled a new morphological matrix based on rhizome anatomy, and used network analyses to establish phylogenetic relationships between fossil and extant members of modern Osmundaceae. We re-analysed the original molecular data to evaluate root-placement support. Finally, we integrated morphological and molecular data-sets using the evolutionary placement algorithm.

**Results:**

*Osmunda pulchella* and five additional Jurassic rhizome species show anatomical character suites intermediate between *Osmundastrum* and *Osmunda.* Molecular evidence for paraphyly is ambiguous: a previously unrecognized signal from spacer sequences favours an alternative root placement that would resolve *Osmunda s.l.* as monophyletic. Our evolutionary placement analysis identifies fossil species as probable ancestral members of modern genera and subgenera, which accords with recent evidence from Bayesian dating.

**Conclusions:**

*Osmunda pulchella* is likely a precursor of the *Osmundastrum* lineage. The recently proposed root placement in Osmundaceae—based solely on molecular data—stems from possibly misinformative outgroup signals in *rbc*L and *atp*A genes. We conclude that the seemingly conflicting evidence from morphological, anatomical, molecular, and palaeontological data can instead be elegantly reconciled under the assumption that *Osmunda* is indeed monophyletic.

**Electronic supplementary material:**

The online version of this article (doi:10.1186/s12862-015-0400-7) contains supplementary material, which is available to authorized users.

## Background

The royal ferns (Osmundales) comprise about 20 extant species currently classified in four genera, i.e. *Osmunda* L., *Osmundastrum* C.Presl, *Leptopteris* C.Presl, and *Todea* Bernh. This small group of ferns is remarkable in many respects and, consequently, has attracted considerable scholarly attention. Its members represent the most primitive of all leptosporangiate ferns [1–4], with features that have been interpreted to be intermediate between Eusporangiatae and Leptosporangiatae [5–7]. Detailed investigations of their anatomy [8–11], cytology and genetic structure [12–23], and evolution [24–34] render the Osmundales one of the most intensively studied groups of ferns. Moreover, in contrast to their rather limited modern diversity, Osmundales have a uniquely rich and diverse fossil record [30, 35] currently considered to include more than 150 species, over 25 genera, and at least three (sub) families. This extensive fossil record has been reviewed in several key works [7, 30, 36, 37] and, recently, been recruited for molecular dating using three contrasting Bayesian approaches (traditional node dating, total-evidence dating, and dating using the fossilized-birth-death approach) [34].

The monophyly of Osmundales and their isolated position as the first diverging lineage within leptosporangiate ferns are firmly established [1, 3, 38, 39]. However, the resolution of systematic relationships within the group—and especially the circumscription of *Osmunda*—continues to remain controversial. Linnaeus established *Osmunda* with three species: *O. regalis* L., *O. claytoniana* L. and *O. cinnamomea* L. [40]. With subsequent descriptions of additional species from East and Southeast Asia [41–44], the genus was subdivided into several subgenera, i.e. *O.* subgenus *Osmunda*, *O*. subgenus *Plenasium* (C.Presl) J.Smith, *O*. subgenus *Osmundastrum* (C.Presl) C.Presl, and *O*. subgenus *Claytosmunda* Y.Yatabe, N.Murak. & K.Iwats. based on combinations of diagnostic morphological and anatomical characters and—more recently—molecular phylogenetic analyses [31, 32, 45]. However, independent lines of evidence based on morphology [11, 46, 47], anatomy [11, 29, 30], palynology [48], hybridization experiments [49–52], and molecular and genetic studies [31, 32, 53–55] have led to divergent opinions on the classification of these taxa. Most controversy has arisen concerning the phylogenetic relationships and taxonomic ranks of *O. cinnamomea* and *O. claytoniana* (please refer to the nomenclatural remark in the methods section for information on the use of taxon names herein).

Early molecular studies aiming to resolve specific relationships between *O. regalis*, *O. claytoniana* and *O. cinnamomea* produced remarkably incongruent results [53, 54]. Isozyme studies eventually demonstrated that *O. claytoniana* is probably more closely related to *O. regalis* than either is to *O. cinnamomea* [55], confirming previous assumptions of early plant anatomists [8, 29, 30]. Subsequent nucleotide sequencing not only provided first robust support for this relationship [31] but, unexpectedly, also placed *Todea* and *Leptopteris* within *Osmunda* as traditionally defined. Consequently, the isolated *O. cinnamomea* at the base of the resulting tree was separated from *Osmunda s.str.* and assigned to its own genus, sister to *Leptopteris* plus *Todea* and the remaining *Osmunda* [32].

Here we describe a new *Osmunda* species based on an exceptionally well-preserved rhizome from the Jurassic of Sweden that combines features diagnostic of *Osmunda* and *Osmundastrum*. A phylogenetic analysis based on a newly assembled morphological character matrix places the new species intermediate between *Osmunda* and *Osmundastrum*, which is incompatible with the recently established paraphyly and resulting classifications. Current notions in phylogenetic research emphasize the significance of integrating morphological with molecular evidence for resolving evolutionary relationships (e.g. [56–58]), especially among ferns [59, 60].

Therefore, we re-analyse the molecular data and integrate morphological and molecular data-sets of fossil and extant Osmundaceae to show that the recently established paraphyly of *Osmunda s.l.* suffers from ambiguous outgroup signals; by contrast, we submit that all evidence can instead be elegantly reconciled assuming *Osmunda s.l.* is indeed monophyletic.

## Results

### Systematic description of the fossil

Order Osmundales Link

Family Osmundaceae Bercht. & C.Presl

Genus *Osmunda* L.

Species *Osmunda pulchella* Bomfleur, G.Grimm & McLoughlin sp. nov.

#### Diagnosis

**Rhizome** creeping or semi-erect. **Stem** with ectophloic-dictyoxylic siphonostele and two-layered cortex. **Pith** entirely parenchymatous. **Xylem cylinder** about 8–12 tracheids (mostly *ca* 0.4 mm) thick, dissected by narrow, complete, immediate leaf gaps, containing about twenty xylem segments in a given transverse section. **Phloem and endodermis** external only. **Inner cortex***ca* 0.5–0.8 mm thick, homogeneous, parenchymatous, containing about ten leaf traces in a given transverse section; **outer cortex***ca* 1.5–2.5 mm thick, homogeneous, sclerenchymatous, containing about 20 leaf traces in a given transverse section. **Leaf traces** in stem oblong, more or less reniform, adaxially concave, endarch with a single protoxylem strand at the point of emergence from stele, diverging at acute angles of *ca* 20–40°; protoxylem strand bifurcating only in outermost cortex or upon departure from stem. **Petiole bases** with adaxially concave vascular strand, one adaxial sclerenchyma band in vascular-strand concavity, parenchymatic cortex, a heterogeneous sclerenchyma ring, and an opposite pair of petiolar wings; **adaxial sclerenchyma** in inner cortex of petiole appearing in the form of a single patch or arch lining the vascular-bundle concavity with homogeneous thickness, differentiating distally into two thickened lateral masses connected by a thin strip, extending proximally only to base of petiole, not into stem; **sclerenchyma ring** of petiole base thicker than vascular bundle, heterogeneous, with a crescentic abaxial cap of thicker-walled fibres in the basal petiole portion differentiating distally into two lateral masses and ultimately into two lateral and one abaxial mass; **petiolar wings** in distal portions containing an elongate strip of thick-walled fibres. **Roots** diarch, usually arising singly from one leaf trace, containing scattered sclerenchyma fibres.

#### Type stratum and age

Mafic pyroclastic and epiclastic deposits informally named the “Djupadal formation” [61]; Pliensbachian (late Early Jurassic).

#### Type locality

Korsaröd lake (55°58’54.6”N, 013°37’44.9”E) near Höör, central Skåne, southern Sweden.

#### Holotype (hic designatus)

A single specimen of permineralized rhizome, sectioned and prepared into six blocks (specimens NRM S069649–S069655) and three microscope slides, including two transverse thin sections (slides NRM S069656 and S069657) and one radial thin section (NRM S069658); all material is curated in the collection of the Department of Palaeobiology, Swedish Museum of Natural History, Stockholm, Sweden.

#### Etymology

The specific epithet *pulchella* (Latin diminutive of *pulchra* =‘beautiful’, ‘fair’) is chosen in reference to the exquisite preservation and aesthetic appeal of the holotype specimen.

#### Description

**Fig. 1:**
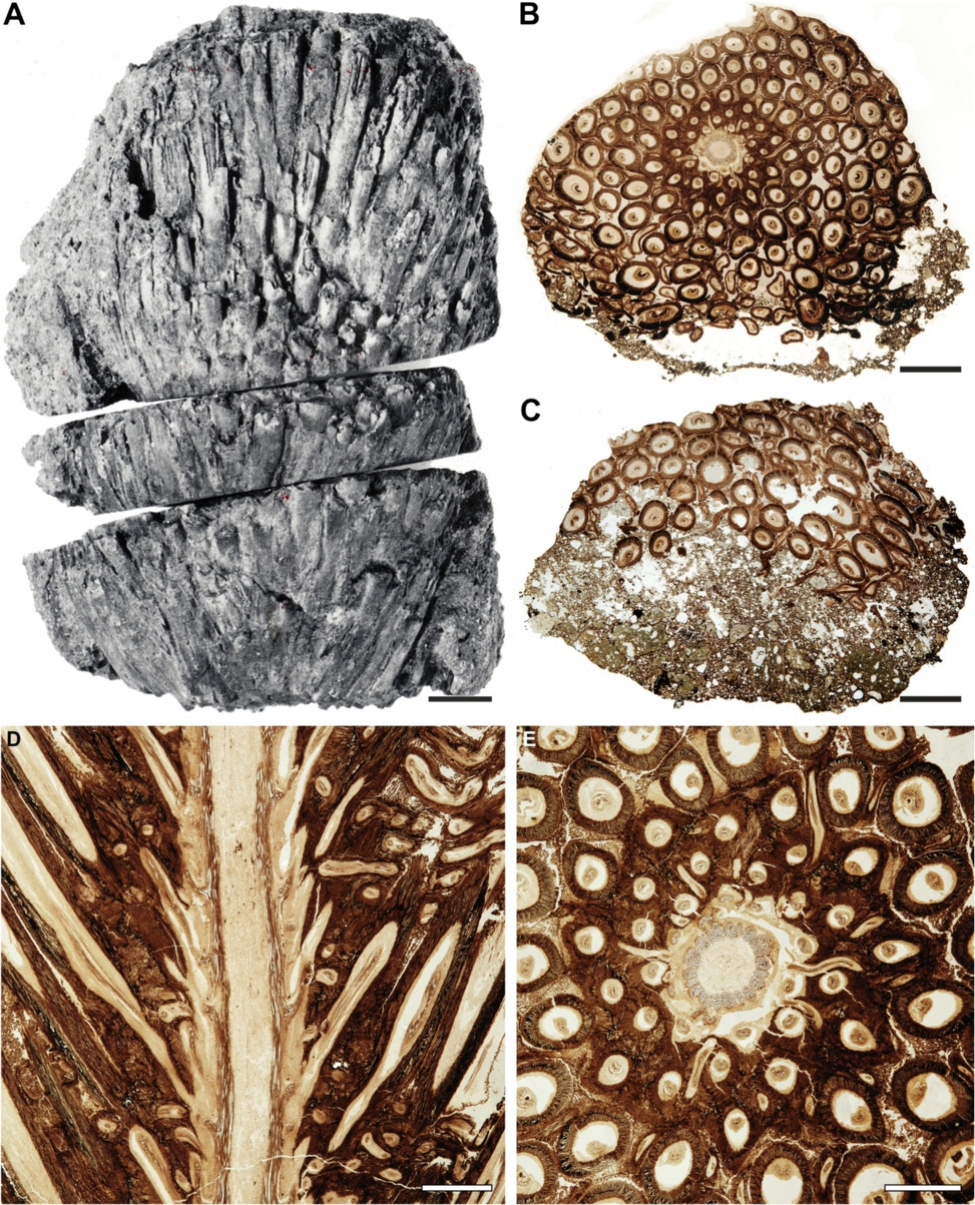
*Osmunda pulchella* sp. nov. from the Lower Jurassic of Skåne, southern Sweden. Holotype. **a** Reproduction of the only available print of the original holotype material prior to preparation, showing the gross morphology of the rhizome. **b**, **c** Transverse sections through center (B: NRM-S069656) and apex (C: NRM-S069657) of the rhizome. **d** Longitudinal section through the rhizome (NRM-S069658). (E) Detail of Fig. 1B. Scale bars: (**a**–**c**) = 5 mm; (**d**, **e**) = 2 mm

The holotype is a calcified rhizome fragment about 6 cm long and up to 4 cm in diameter (Fig. 1a–c). It consists of a small central stem that is surrounded by a compact mantle of helically arranged, persistent petiole bases and interspersed rootlets (Fig. 1b, e). The rootlets extend outwards through the mantle in a sinuous course almost perpendicular to the axis, indicating low rhizomatous rather than arborescent growth; the asymmetrical distribution of roots in longitudinal sections of the rhizome (Fig. 1d) points to a creeping habit.

**Fig. 2:**
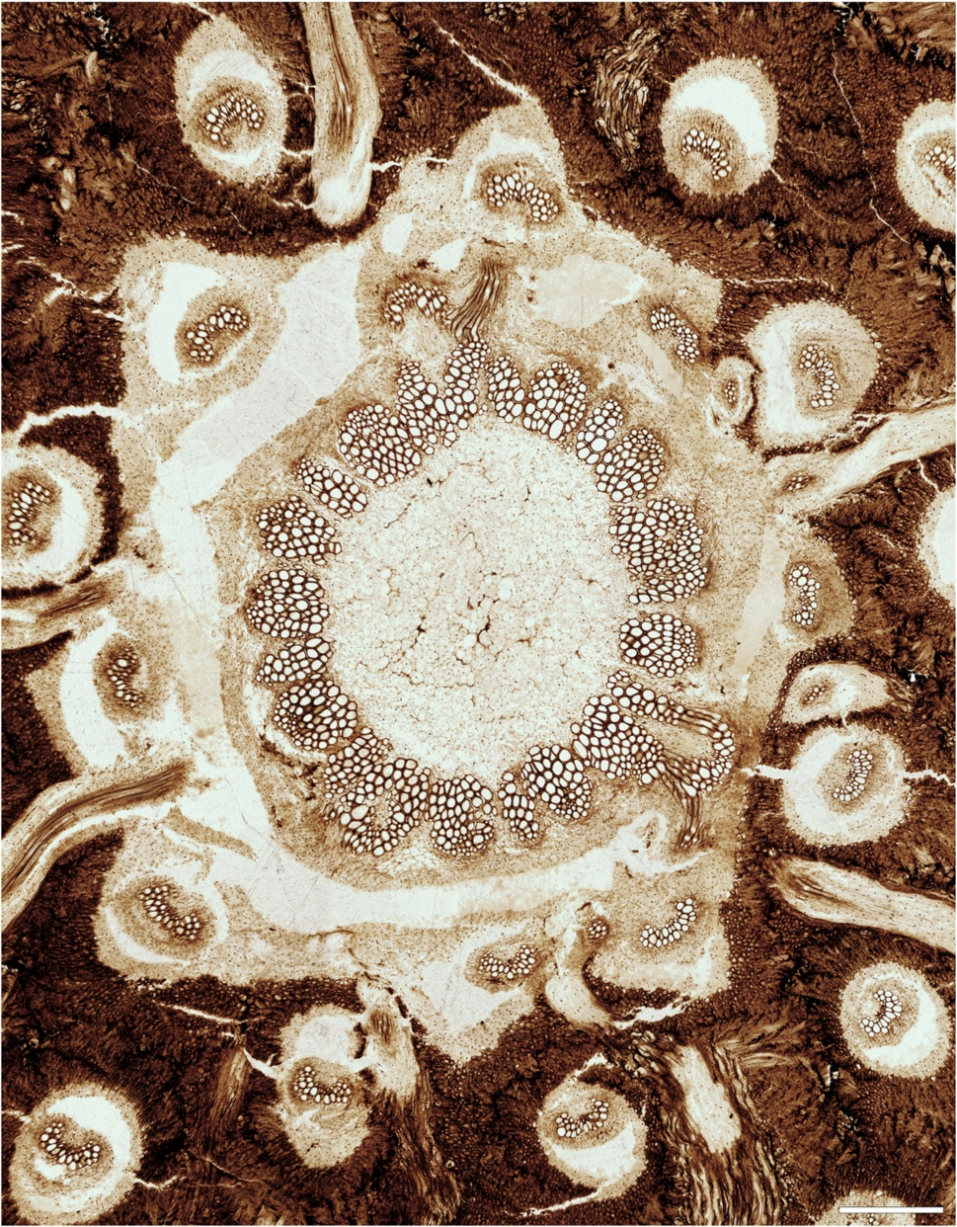
*Osmunda pulchella* sp. nov. from the Lower Jurassic of Skåne, southern Sweden. Transverse section through the stem (NRM-S069656). Scale bar = 500 μm

**Fig. 3:**
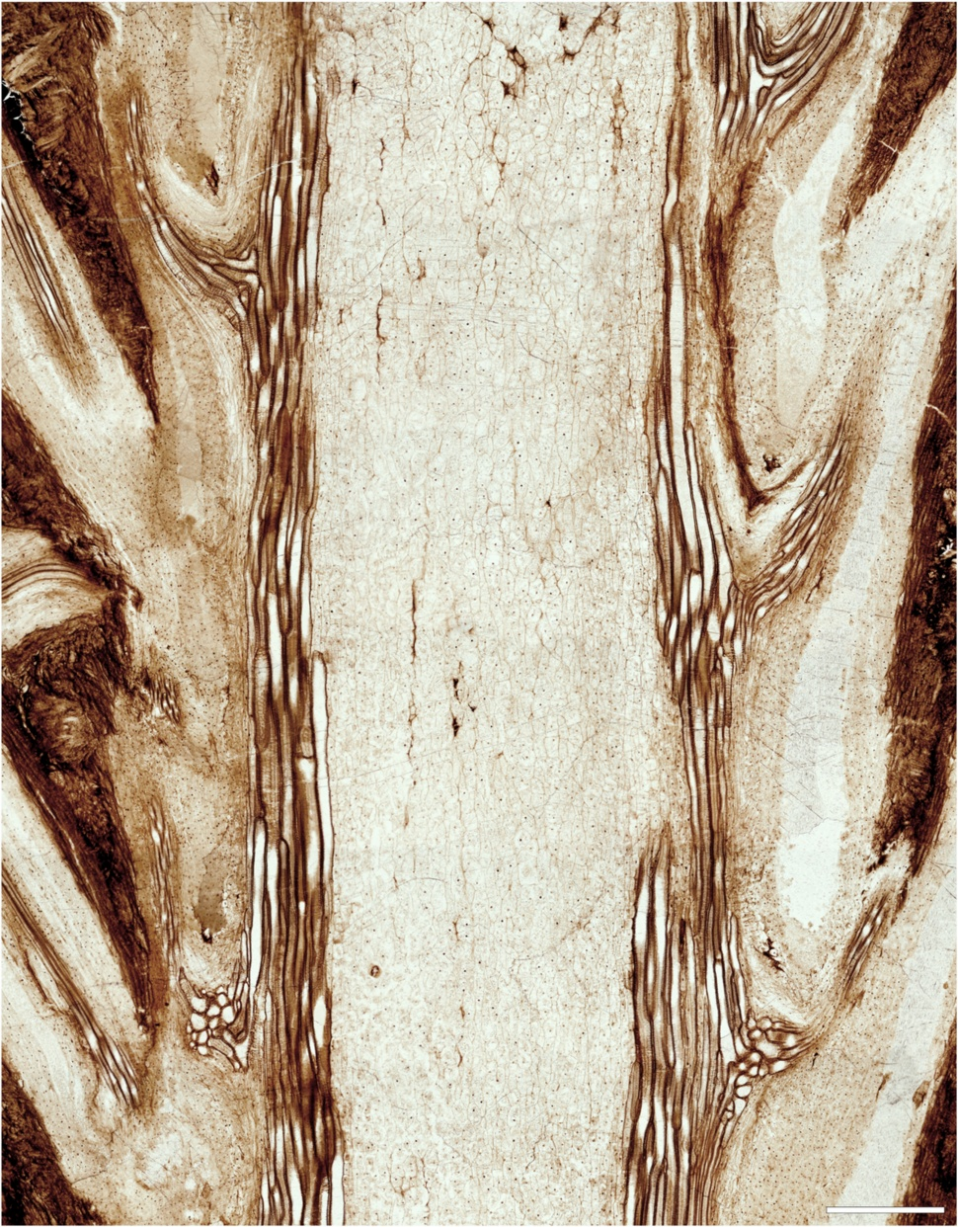
*Osmunda pulchella* sp. nov. from the Lower Jurassic of Skåne, southern Sweden. Radial longitudinal section through the stem (NRM-069658). Scale bar = 500 μm

**Fig. 4:**
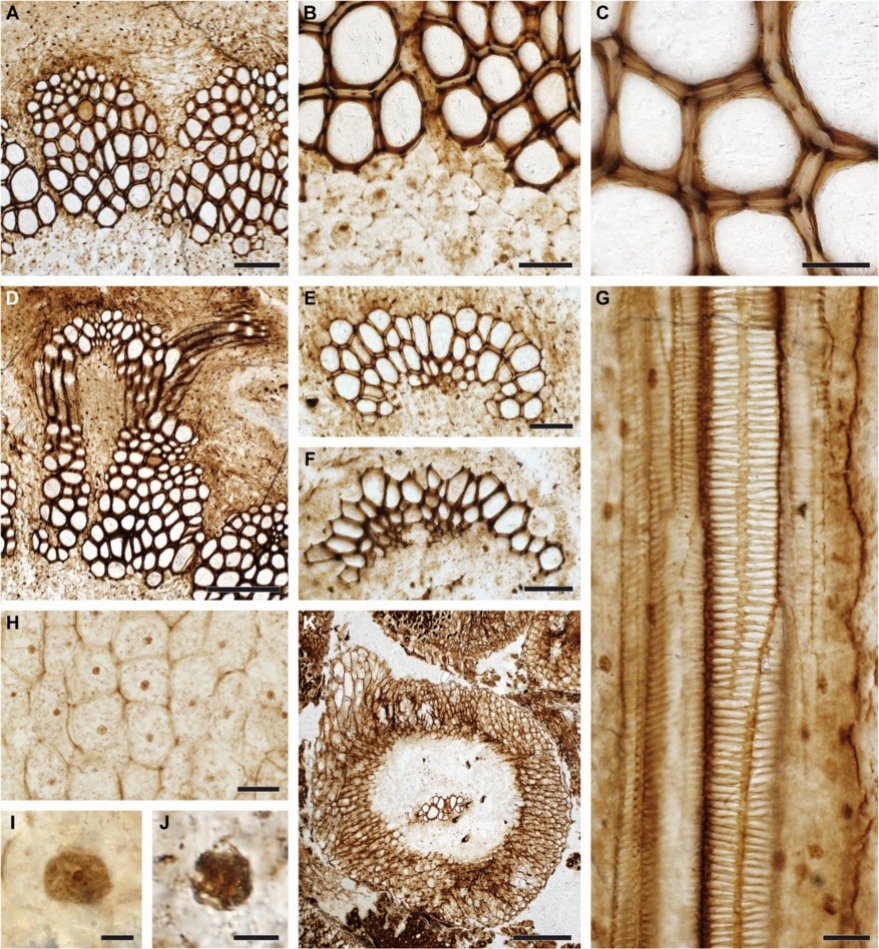
Anatomical and cytological details of *Osmunda pulchella* sp. nov. from the Lower Jurassic of Skåne, southern Sweden (A–F, I: NRM-S069656; G, H, J, K: NRM-S069658). **a** Detail showing pith parenchyma (bottom), stelar xylem cylinder dissected by complete leaf gaps, triangular section of phloem projecting into leaf gap, and parenchymatous inner cortex (top); note mesarch leaf-trace protoxylem initiation in the stelar xylem segment on the right. **b** Detail of (**a**) showing peripheral pith parenchyma and stem xylem. **c** Detail of stem xylem showing tracheid pitting. **d** Endarch leaf trace emerging from the stele and associated with a single root. **e** Leaf trace in the inner cortex of the stem showing single, endarch protoxylem cluster. **f** Leaf trace immediately distal to initial protoxylem bifurcation in the outermost cortex of the stem. **g** Root vascular bundle showing well-preserved scalariform pitting of metaxylem tracheids. **h** Well-preserved pith parenchyma showing membrane-bound cytoplasm with cytosol particles and interphase nuclei containing nucleoli. **i**, **j** Nuclei with conspicuous nucleoli (in interphase: I) or with distinct chromatid strands (in prophase: J). **k** Transverse section through root showing diarch vascular bundle, parenchymatous inner cortex with isolated fibre strands, and prominent fibrous outer cortex. Scale bars: (**a**) = 100 μm; (**b**, **e**, **f**, **h**) = 50 μm; (**c**, **g**) = 25 μm; (**d**) = 200 μm; (**i**, **j**) = 5 μm; (**k**) = 250 μm

**Fig. 5:**
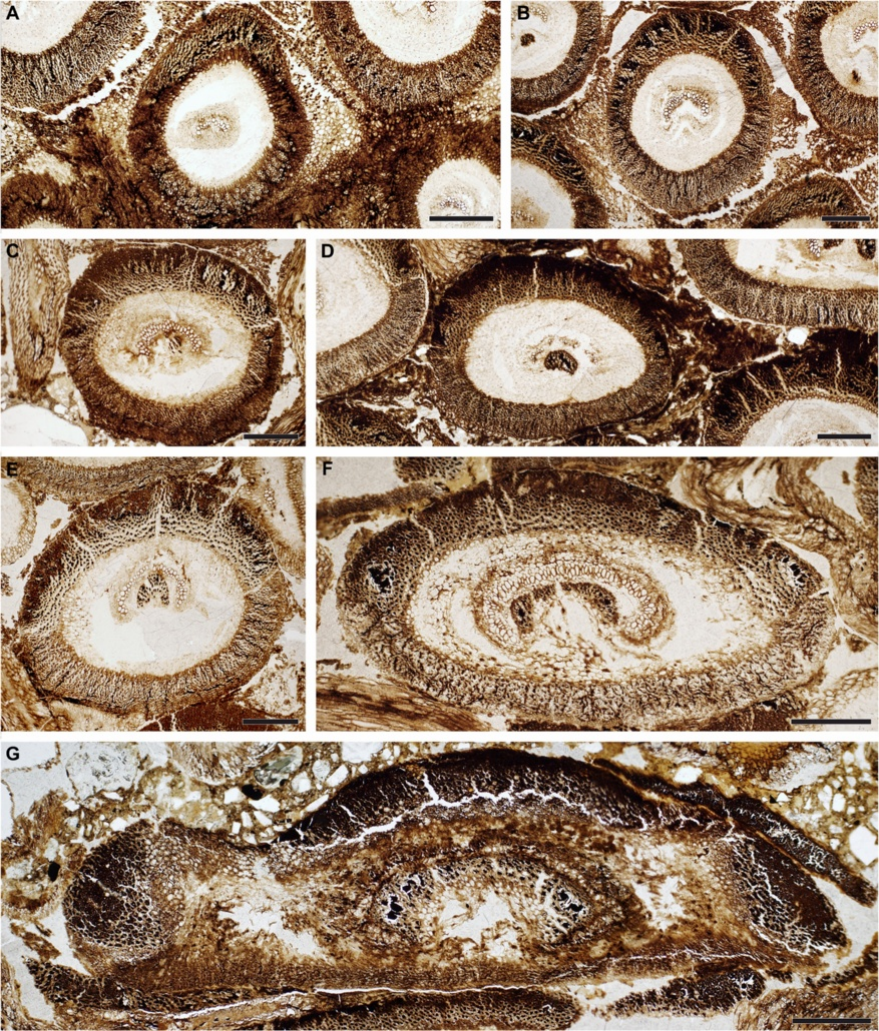
Basal to distal sections of petiole bases of *Osmunda pulchella* sp. nov. from the Lower Jurassic of Skåne, southern Sweden (NRM-S069657), showing successive stages of petiole-base differentiation. **a** Parenchymatous inner cortex and petiolar wings. **b**–**e** Development of an abaxial arch of thick-walled fibres in the sclerenchyma ring. **c** Appearance of a sclerenchyma patch in the bundle concavity. **d** Appearance of a sclerenchyma mass in the petiolar wing. **f** Sclerenchyma ring with two prominent lateral masses of particularly thick-walled fibres. **g** Collapsed outermost petiole (note rock matrix above) showing sclerenchyma ring with one abaxial and two lateral masses of particularly thick-walled fibres, and elongate sclerenchyma strips (e.g. bottom, right) isolated from degraded stipular wings of adjacent petioles. Scale bars = 500 μm

The stem is *ca* 7.5 mm in diameter, and consists of an ectophloic-dictyoxylic siphonostele surrounded by a two-layered cortex (Figs. 1d, e, 2, 3, 7a). The pith is *ca* 1.5 mm in diameter and entirely parenchymatous (Fig. 2). A thin region at the outermost periphery of the pith consists of a few rows of parenchyma cells that are considerably more slender (*ca* 20–30 μm wide) than those in the central portion of the pith (usually ≥ 50 μm wide; Figs. 3, 4a, b). Furthermore, cell walls in some regions of the pith periphery may be thicker and more clearly visible than in the centre (Figs. 3, 4b). However, there is no evidence for the presence of an internal endodermis or internal phloem. Given that endodermal layers are recognizable in the stem and petiole cortices (e.g. Fig. 5f), we are certain that the absence of an internal endodermis is an original feature, and not the result of inadequate preservation. The xylem cylinder is *ca* 0.4 mm and *ca* 8–12 tracheids thick, and dissected by narrow, mostly complete, immediate leaf gaps into about 20 xylem segments in a given transverse section. The phloem forms an entire ring around the stele; it is most easily recognizable opposite a leaf gap, where it forms a narrow wedge-shaped patch of large, thin-walled cells that projects slightly towards the gap in transverse section (Figs. 3, 4a).

The cortex of the stem is bi-layered (Figs. 1e, 2, 3, 7a). The inner layer is *ca* 0.5–0.8 mm thick, consists entirely of parenchyma, and contains about ten leaf traces in a given transverse section (Fig. 2). The outer cortex is considerably thicker (*ca* 1.5–2.5 mm thick), and consists entirely of homogeneous sclerenchymatic tissue (Figs. 1e, 2, 3). Abundant leaf traces (about 20 in a given transverse section; e.g. Fig. 2) and rootlets traversing the outer cortex (Figs. 1c, d2) appear to have altered the original orientation of the sclereids, resulting in a somewhat patchy appearance of the outer cortical tissue (Fig. 2).

Phyllotaxy of the stem is helical with apparent contact parastichies of 8 and 13 (Fig. 1b, e). Leaf-trace formation begins with the appearance of a single protoxylem strand in an eccentric position (about two-thirds to three-quarters distance from the pith; Fig. 4a) in a stelar metaxylem segment. Distally, the protoxylem becomes associated with an increasing amount of parenchyma on its adaxial side (making it effectively endarch for the rest of its course), first occupying only the centre of the segment (resulting in an O-shaped xylem segment), then connecting with the pith (resulting in a U-shaped xylem segment), and ultimately forming the complete, narrow leaf gap with the departure of the trace. Departing leaf traces are oblong, only slightly curved adaxially, *ca* 300–350 μm wide and two to four tracheids (*ca* 80–100 μm) thick (Figs. 2, 4d); they diverge from the axis at angles of *ca* 20–40° (Figs. 1a, 3).

In its course through the stem, a leaf-trace vascular bundle becomes enveloped by increasing layers of tissue through which it passes successively: first by phloem and endodermis from the stele upon entering the inner cortex; by a sheath of parenchyma from the inner cortex as it enters the outer cortex (Figs. 2, 3); and finally by a cylindrical sclerenchyma sheath from the outer cortex as it departs from the stem (Fig. 1e). The initial bifurcation of the leaf-trace protoxylem occurs in the outermost portion of the cortex or in the petiole base (Fig. 4e, f).

**Fig. 6:**
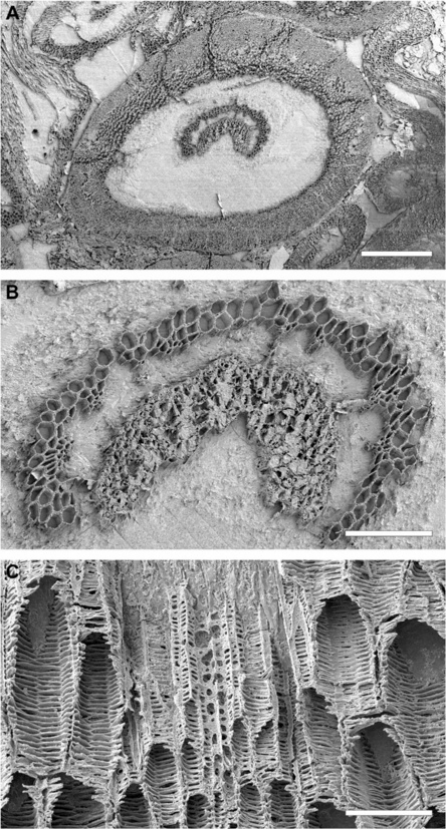
Details of petiole-base anatomy of *Osmunda pulchella* sp. nov. from the Lower Jurassic of Skåne, southern Sweden, revealed via scanning electron microscopy. **a** Distal cross-section through a petiole. **b** Detail of (**a**) showing vascular strand with about eight endarch protoxylem bundles and sclerenchyma mass lining the vascular-strand concavity. **c** Detail showing helical wall thickening of protoxylem strands (center) compared to multiseriate scalariform wall thickenings of metaxylem tracheids in a petiole vascular bundle (oriented with adaxial side facing upwards). Scale bars: (**a**) = 1 mm; (**b**) = 100 μm; (**c**) = 50 μm

In the inner cortex of the petiole, thick-walled fibres appear in the form of a small irregular mass adaxial to the vascular bundle (Fig. 5c, d). This mass develops distally into a thick band lining the bundle concavity (Figs. 5e, 6a, b), and may further differentiate into two lateral masses connected only by a rather thin strip (Fig. 5f, g). Apart from the sclerenchyma inside the vascular-bundle concavity, the inner cortex of the petiole consists entirely of parenchyma.

**Fig. 7:**
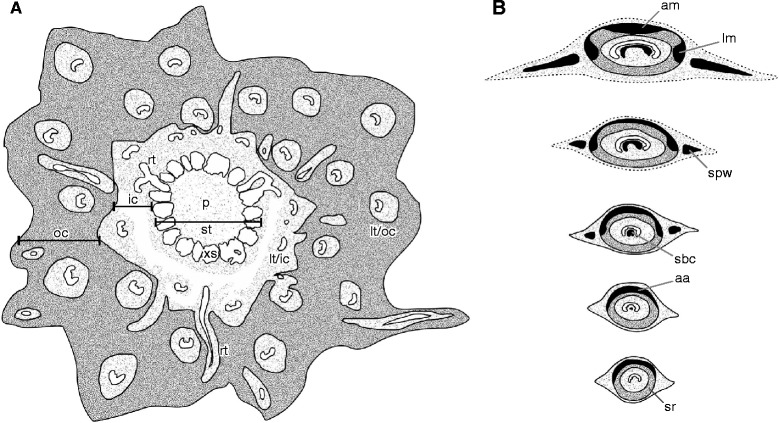
Schematic drawings showing diagnostic anatomical characters of *Osmunda pulchella* sp. nov. from the Lower Jurassic of Skåne, southern Sweden. **a** Stem cross section. **b** Successive cross sections of basal (bottom) to distal (top) petiole portions. Xylem in white; parenchyma in light-grey; sclerenchyma in dark-grey; sclerenchyma with particularly thick-walled fibres in black; oc = outer cortex; ic = inner cortex; st = stele; p = pith; xs = stelar xylem segment; lt/ic = leaf trace in inner cortex; lt/oc = leaf trace in outer cortex; rt = root trace; sr = sclerenchyma ring; aa = abaxial arch of thick-walled fibres; sbc = sclerenchyma mass in bundle concavity; spw = sclerenchyma mass in petiole wing; lm = lateral masses of thick-walled fibres; am = abaxial mass of thick-walled fibres

The sclerenchyma cylinder of the petiole has an even thickness that increases from about 300 μm near the petiole base to *ca* 500 μm distally. Its composition is heterogeneous: near the petiole base, it contains a crescentic, abaxial arch of particularly thick-walled fibres (Figs. 1e, 5, 6, 7b); distally, this arch begins to develop two lateral masses (Figs. 5d–f, 7b) and ultimately two lateral masses and one abaxial arch of thick-walled fibres whose lumina are more-or-less entirely occluded (Figs. 5g, 7b).

The petiole bases are flanked by a pair of stipular wings that consist initially of parenchyma only. As the wings grow wider in more distal portions, they develop a patch of thick-walled fibres (Figs. 5d, 7b) that forms an entire, elongate strip (Figs. 5f, g7b). The parenchymatic ground tissue of the stipular wings is well-preserved only in the innermost regions of the mantle (Fig. 1b, c, e); outwards, it appears to be either increasingly degraded or to have been removed by pervasive rootlet growth. In the outermost portions of the mantle, all that remains of the stipular wings are usually just the isolated, elongate strips of thick-walled fibres interspersed between petioles and rootlets (Fig. 5g).

Each leaf trace is usually associated with a single rootlet that diverges laterally at the point of departure from the stele. The rootlets typically measure about 0.5 mm in diameter, contain a diarch vascular bundle, parenchymatic ground tissue with interspersed sclerenchymatic fibres, and a sclerenchymatic outer cortical layer.

The holotype of *O. pulchella* has a phenomenal preservational quality revealing cellular and subcellular detail (Figs. 2, 3). Tracheids have exquisitely preserved wall thickenings, which are scalariform in metaxylem (Figs. 4cg, 6c) and annular to helical in protoxylem cells (Fig. 6c). Most parenchyma cells contain preserved cellular contents (Figs. 2, 3), including nuclei (Fig. 4h–j), membrane-bound cytoplasm (Fig. 4h), and cytosol granules [62]. Some parenchyma cells, especially those adjacent to xylem bundles in roots and leaf traces, contain varying amounts of discrete, smooth-walled, spherical or oblate particles *ca* 1–5 μm in diameter that have been interpreted as putative amyloplasts [62]. Cell nuclei measure *ca* 10 μm in diameter, and contain nucleoli and, in a few cases, unravelled chromosomes (Fig. 4i, j). Chromatid strands have a diameter of 0.3–0.4 μm (Fig. 4j).

### Phylogenetic analyses

#### Phylogenetic relationships among fossil and modern members of the Osmundaceae based on rhizome anatomy

**Fig. 8:**
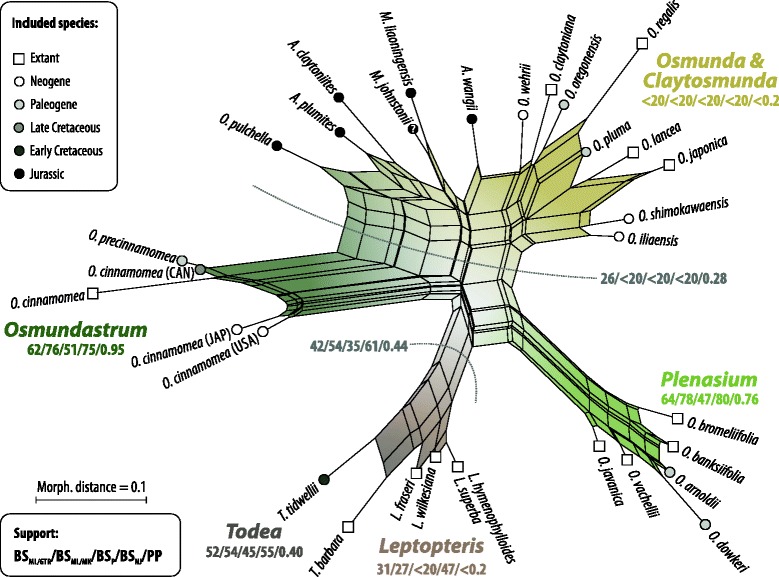
Neighbour-net showing phylogenetic relationships among fossil and extant members of modern Osmundaceae inferred from a morphological distance matrix based on rhizome anatomy. Edge (branch) support from bootstrapping (BS) and Bayesian inference (posterior probability, PP) is annotated for modern genera and subgenera, and selected bipartitions. Further abbreviations: BS_ML/GTR_ = maximum likelihood (ML) BS support using a general-time reversible transformation model; BS_ML/MK_ = BS support using Lewis’ one-parameter model [146]; BS_P_ = parsimony BS support; BS_NJ_ = neighbour-joining BS support; see Additional file 1 [ESA]

The phylogenetic network based on pairwise distances inferred from a matrix including 23 rhizome anatomical characters resolved five major species groups: (1) extant species of *Leptopteris* and *Todea* together with *T. tidwellii* Jud, G.W.Rothwell & Stockey from the Lower Cretaceous of North America; (2) all extant species of *Osmunda* subgenus *Plenasium* together with *O. arnoldii* C.N.Mill. and *O. dowkeri* (Carruth.) M.Chandler from the Paleogene of North America and Europe; (3) all species of subgenus *Osmunda sensu* Miller, i.e. species of the extant subgenera *Osmunda sensu* Yatabe *et al.* and *Claytosmunda* together with several Paleogene and Neogene species; (4) all Jurassic rhizome species, including *O. pulchella*; and (5) all extant and fossil members of *Osmundastrum* (*O. cinnamomea* and *O. precinnamomea*) (Fig 8). Corresponding bipartitions, which would define clades in an accordingly rooted phylogram, were found in the bootstrap replicate tree sample and the Bayesian sampled topologies with varying frequency. *Osmunda* subgenus *Plenasium* (BS = 47–80; PP = 0.76) and *Osmundastrum* (BS = 51–76; PP = 0.95) received best support*,* whereas support values for the other groups were generally low (BS ≤ 55; PP ≤ 0.43). The Jurassic species bridge the morphological gap between *Osmundastrum* and *Osmunda* subgenus *Osmunda sensu* Miller, with *O. pulchella* being the species closest to *Osmundastrum*. A hypothetical clade comprising *Osmunda* subgenus *Osmunda sensu* Miller and the Jurassic *Osmunda* species would receive BS up to 28 and PP of 0.28.

Especially remarkable is the diversification of subgenus *Osmundastrum* as revealed by our independent coding of the individual fossil records from Neogene [29, 63], Paleogene [29], and Cretaceous deposits [64]; the individually coded fossil and extant representatives assigned to *O. cinnamomea* show greater morphological disparity than expressed between the separate species of any other subgenus and genus.

#### Merging fossil and extant taxa into a molecular backbone topology

**Fig. 9:**
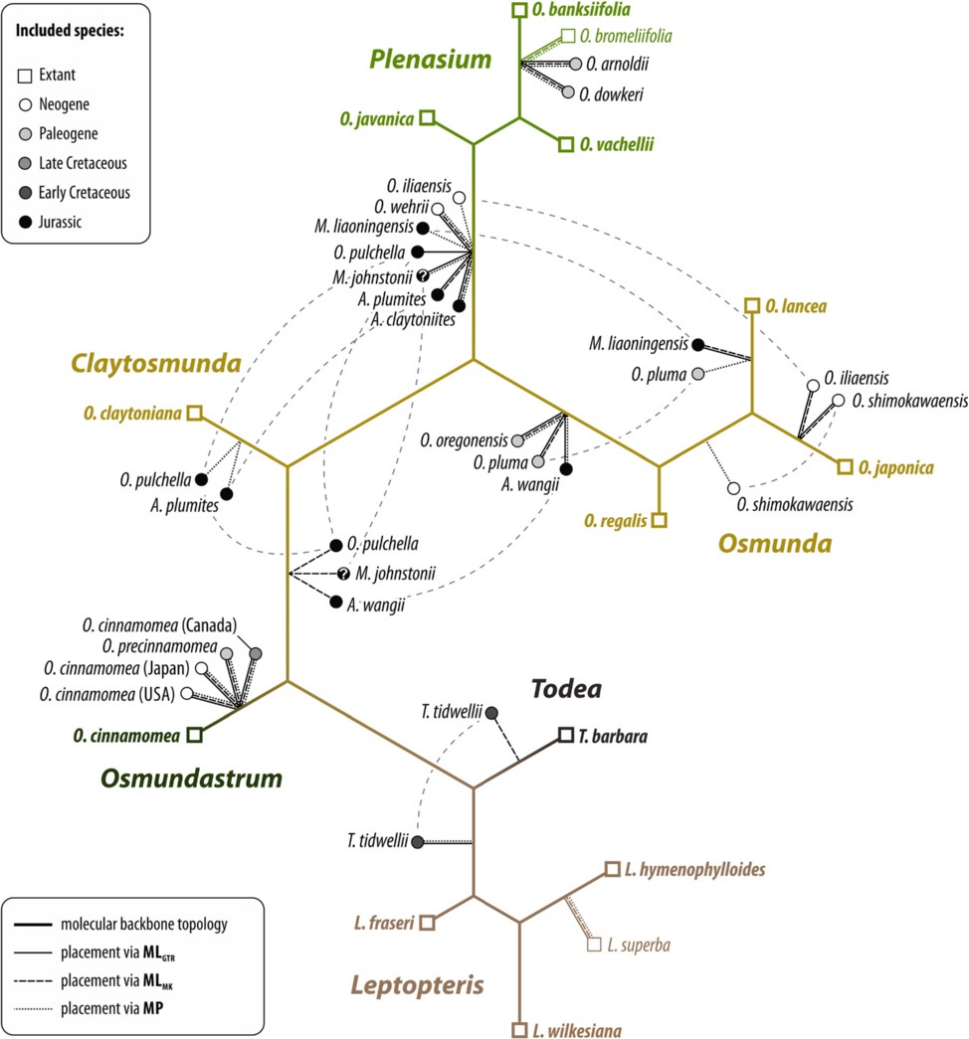
Placement of fossil and extant members into the specified backbone topology of modern Osmundaceae inferred from molecular data of Metzgar *et al.* [32] using the evolutionary placement algorithm [147] (see Additional file 1 [ESA]) and three different character-weighting schemes. Dashed light-grey lines indicate weighting-scheme-dependent position swaps of taxa. Abbreviations: ML_GTR_ = weighting scheme optimized under a general-time reversible transformation model; ML_MK_, weighting scheme optimized under Lewis’ (2001) model; MP = weighting scheme under parsimony

Of all taxa placed via the evolutionary placement algorithm (EPA; Fig. 9), *Osmunda pulchella* is the species that is most incongruently placed between the different weighting schemes: Using parsimony-based character weights, the EPA places *Osmunda pulchella* at the root of *Claytosmunda*, whereas it is placed either between *Osmundastrum* and the remaining *Osmunda s.str.* or at the root of the *Plenasium* clade using model-based character weights (Fig. 9). Single position swaps also occur in most of the other Jurassic species [*Ashicaulis* (= *Millerocaulis sensu* Vera) *plumites* N.Tian & Y.D.Wang, *A.* (= *Millerocaulis sensu* Vera) *wangii* N.Tian & Y.D.Wang, *Millerocaulis johnstonii* Tidwell, Munzing & M.R.Banks, *M. liaoningensis* Wu Zhang & Shao-Lin Zheng] and in *O. pluma* C.N.Mill., *O. iliaensis* C.N.Mill., *O. shimokawaensis* M.Matsumoto & H.Nishida, and *Todea tidwellii*. Except for *Todea tidwellii* (placed at the root of either *Leptopteris* or *Todea*), all swaps occur within the *Osmunda s.l.* sub-tree. Swaps among the Jurassic species mostly involve placements at the root of the *Plenasium* sub-tree, the subgenus *Osmunda* sub-tree, and at the branch between *Osmundastrum* and the remaining *Osmunda. Osmunda shimokawaensis* and *O. iliaensis* are variably placed within the *O. lancea* Thunb.*–O. japonica* Thunb. sub-tree.

By contrast, fixed placements congruent over all three weighting schemes employed occur in: fossil members of *Osmundastrum* (all at the *O. cinnamomea* branch); *Ashicaulis* (= *Millerocaulis sensu* Vera) *claytoniites* Y.M.Cheng and *O. wehrii* C.N.Mill. (at the root of the *Plenasium* sub-tree); *O. arnoldii*, *O. bromeliifolia* (C.Presl) Copel., and *O. dowkeri* (all at *O. banksiifolia* branch); *O. oregonensis* (C.A.Arnold) C.N.Mill. (at the root of subgenus *Osmunda*), and *L. superba* (at the branch of *L. hymenophylloides*).

#### Re-visitation of the outgroup-inferred Osmundaceae root

**Fig. 10:**
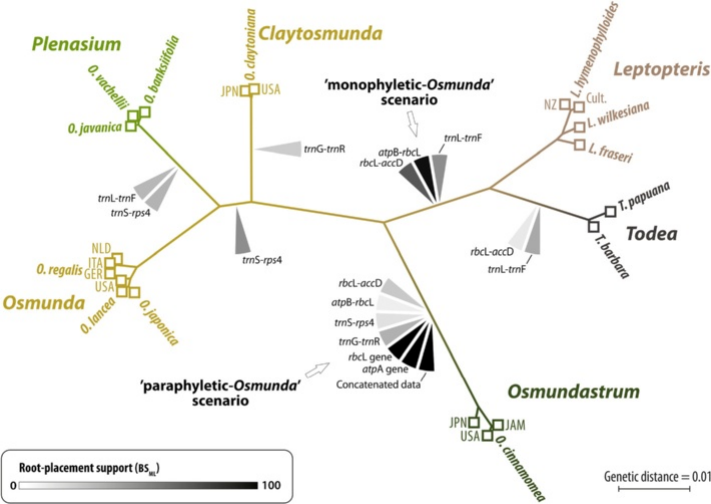
Phylogenetic tree, optimized under maximum likelihood (ML), showing unambiguously resolved relationships among extant Osmundaceae and the conflicting root-placement (outgroup-inferred) signals from individual gene regions. Based on the molecular matrix compiled and employed by Metzgar *et al.* [32]. All backbone branches received full maximum-likelihood bootstrap support (BS_ML_ = 100) based on the concatenated data; support for leaf-branches not shown (see Additional file 1 [ESA])

The gene jackknifing and single-gene analyses reveal ambiguity concerning the position of the Osmundaceae root in the data of Metzgar *et al.* [32] (Fig. 10). As in the original analysis [32], support for backbone branches is effectively unambiguous based on the concatenated data, and the outgroup-inferred root is placed between *Osmundastrum* and the remainder of the family, resolving the traditional genus *Osmunda* (*Osmunda s.l.*) as a grade (‘paraphyletic *Osmunda* scenario’). The signal for this root placement stems from the two coding plastid gene regions (*atp*A and *rbc*L). In the more (but not most) variable spacer regions (*atp*B-*rbc*L, *rbc*L-*acc*D, and *trn*L-*trn*F to a lesser degree), however, a competing signal is found resolving *Osmunda s.l.* as a clade (‘monophyletic *Osmunda* scenario’)*.* The most variable non-coding spacer regions (*trn*G-*trn*R; *rps*4-*trn*S; and *trn*L-*trn*F to some degree) provided only ambiguous signals including potential outgroup-branch placements deep within the *Leptopteris-Todea* and *Osmunda* sub-trees or showed a preference for an *Osmundastrum-Leptopteris-Todea* clade as sister to *Osmunda s.str.*

The gene-jackknifing results showed that the exclusion of either one or both coding regions (*atp*A, *rbc*L)—which together account for 33 % of distinct alignment patterns in the concatenated matrix—decreased support for the split leading to an *Osmunda* grade with *Osmundastrum* resolved as sister to the remainder of the family, whereas the support for the alternative of an *Osmunda* clade or an *Osmundastrum-Leptopteris-Todea* clade was increased. In the case of *O. (Claytosmunda) claytoniana*, the genetic data provided a coherent signal, with all plastid regions preferring a subgenus *Osmunda sensu* Yatabe *et al.–Plenasium* clade over the alternatives of a subgenus *Osmunda sensu* Miller or *Claytosmunda-Plenasium* clade. The gene-knifing had no measurable effect (BS_ML_ = 98–100). The problem concerning the placement of the root can also be illustrated in the form of a neighbour-net splits graph based on genetic, uncorrected p-distances [see Additional file 2: Figure S1 in Electronic Supplementary Archive (ESA)].

#### Placement of Osmunda pulchella within the two molecular backbone topologies

**Fig. 11:**
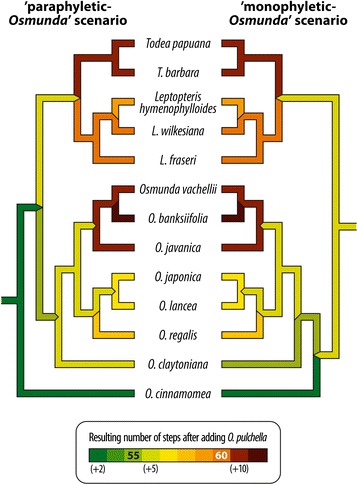
Diagram illustrating the most parsimonious phylogenetic placement of *Osmunda pulchella* within the molecular-based topology under both ingroup rooting scenarios. Left, outgroup-inferred coding-gene-based root (Fig. 10 [31, 32]). Right, alternative rooting (this study)

Optimization of the anatomical characters on two specified backbone topologies inferred from the different rooting scenarios (‘monophyletic *Osmunda*’ *vs* ‘paraphyletic *Osmunda*’ scenario) required 53 steps under parsimony (Fig. 11). Inserting *Osmunda pulchella* into the ‘paraphyletic *Osmunda* scenario’ tree, its most parsimonious placement based on anatomical characters is alternatively (1) at the most basal position as sister to all extant Osmundaceae, (2) as sister to *O. cinnamomea*, or (3) as sister to a putative *Leptopteris-Todea-Osmunda s.str.* clade. In the ‘monophyletic *Osmunda* scenario’ tree, by contrast, the most parsimonious placement of *O. pulchella* is as sister to *O. cinnamomea*. In both trees, the least parsimonious positions of *O. pulchella* are within the *Todea*-*Leptopteris* clade or at the root of or within the *Plenasium* sub-tree.

## Discussion

Many phylogenetic hypotheses have been proposed for Osmundaceae over the past decades, each differing in terms of the data matrices employed and the taxon relationships obtained [30–34, 37, 54, 55, 65–68]. The evolutionary history of the family is clearly difficult to resolve based on the characters of the extant representatives alone. Several researchers have, thus, urged the incorporation of fossil data to assist phylogenetic reconstructions of Osmundaceae [30, 34, 37, 65] and of ferns in general [59, 60, 69, 70].

In the following sections, we (1) place the new fossil species in the broader context of the Mesozoic–Cenozoic fossil record of Osmundaceae; (2) explain the rationale for the assignment of this and other fossil species to an (initially) extant genus; (3) examine the systematic relationships between *Osmunda pulchella* and other fossil and extant species of modern Osmundaceae; (4) provide a critical re-evaluation of the evidence for generic separation of *Osmundastrum* and the paraphyly of *Osmunda s.l.*; and (5) discuss the critical significance of *O. pulchella* for the systematic classification and evolutionary history of modern Osmundaceae.

### Osmundaceae in the regional fossil flora

*Osmunda pulchella* sp. nov. is among the earliest fossil *Osmunda* rhizomes yet known, and the first such find from the Mesozoic of Europe. Whole plants are rarely fossilized, so identification of fossils depends on recognizing diagnostic characters in various dispersed organs. Moreover, some isolated organs can only be identified to taxa under special preservational states (e.g. where anatomical details are retained). Fossil evidence for Osmundaceae occurs in three main forms: (1) permineralized axes with vascular, cortical and petiolar anatomy characteristic of the family; (2) compressions and impressions of foliage (either fertile or sterile); and (3) dispersed spores with sculptural characters typical of fertile macrofossil or extant representatives of the family.

Permineralized osmundaceous axes have a long-ranging and geographically broad fossil record extending back to at least the Permian of both hemispheres [36, 37, 71]. These fossils are highly informative of the anatomical evolution of the group since they preserve the three-dimensional architecture of axial tissues and the surrounding sheath of petioles [30]. They provide further information on osmundacean ecology, since the excavations or coprolites of various invertebrates are commonly preserved within the cortical tissues or petiole sheath [72]. However, occurrences of permineralized axes are generally restricted to sedimentary rocks with a high proportion of volcanogenic components. Free silica and, in some cases, carbonate ions are liberated in particularly high concentrations from the breakdown of glass and unstable calc-silicate minerals, especially in sediments derived from mafic to intermediate volcanic terrains [73]. These ions preferentially link to free hydrogen bonds of holocellulosic complexes in buried plant matter, entombing the original cell walls in opaline silica, quartz, or calcite. The exceptional circumstances of such preservational conditions mean that permineralized osmundaceous stems have a patchy record (see [36] and [74] for summaries of occurrences). Although axes are known from both older (Permian: [26]) and younger (Cenozoic: [28, 75, 76]) rocks in the region, no osmundaceous rhizomes have thus far been reported from the Mesozoic of Europe.

Compressions and impressions of foliage can only be assigned to Osmundaceae with confidence where details of the sori arrangement or sporangial annulus architecture can be resolved [7]. Remains of such fertile fronds are variously assigned to *Osmundopsis* T.M.Harris, *Todites* Seward, *Anomopteris* Brongn., *Cacumen* Cantrill & J.A.Webb*, Cladotheca* T.Halle*,* and *Osmunda* [7, 77–79] and possibly *Damudopteris* D.D. Pant & P.K. Khare and *Dichotomopteris* Maithy [80, 81]. Morphologically similar sterile fronds are typically assigned to *Cladophlebis* Brongn., although not all forms referred to this fossil genus are necessarily osmundacean. Collectively, the record of fossil osmundacean foliage matches that of the rhizomes, extending from the Permian to Cenozoic and being distributed on all continents [30, 82–85]. Foliage referable to *Todites* or *Cladophlebis* is widespread in the Mesozoic of Europe and is extensively represented in Rhaetian to Early Jurassic strata of southern Sweden [86–90].

Spores attributed to Osmundaceae found *in situ* within fossil sporangia or dispersed within sediments are spherical to triangular and typically bear irregularly arranged grana, bacula or pila of variable form and size. More rarely, the spore surface is scabrate or laevigate. When found dispersed, such spores are most commonly assigned to *Osmundacidites* Couper, although some have been attributed to *Baculatisporites* Pflug & P.W.Thomson, *Cyclobaculisporites* D.C.Bhardwaj, *Todisporites* Couper, *Punctatisporites* A.C.Ibrahim, *Leiotriletes* R.Potonié & Kremp, or *Triquitrites* L.R.Wilson & E.A.Coe [78]. Such spores match the record of osmundaceous foliage and permineralized axes in ranging from the Permian to present, and occurring in considerable abundance during the Mesozoic [78]. *Osmundacidites wellmanii* (Couper) Danzé-Corsin & Laveine is one of the dominant spore types recovered from sediments surrounding the fossil rhizome studied herein [62] attesting to the strong representation of this family in the flora of the Korsaröd area during the Pliensbachian. Moreover, *Osmundacidites* and *Baculatisporites* species are common elements of palynofloras recovered from the uppermost Triassic to Middle Jurassic strata throughout southern Sweden [91–95], indicating that the family had an important role in the ecology of the herbaceous stratum of the regional mid-Mesozoic vegetation. Osmundaceae underwent a notable decline in both relative diversity and abundance accompanying the rise of the angiosperms in the Cretaceous [96, 97] and this trend appears to have persisted through the Cenozoic resulting in the family’s low representation and, for some genera, relictual distribution today [85].

### Assignment to *Osmunda*

There is no standard rule in palaeontology deciding whether fossil remains can (or should) be assigned to extant genera or species [79, 98, 99]. In each case, this decision must be taken individually after careful evaluation of the completeness of preservation (i.e. the degree of comparability with extant taxa) and of the diagnostic significance of the preserved morphological characters available for comparison.

Historically, permineralized rhizomes similar to those of extant Osmundaceae have been routinely placed in fossil genera, such as *Osmundites* Unger [27, 28, 100]. Based on a comparative study of fossil rhizomes and extant taxa, however, Chandler [75] concluded that *Osmundites dowkeri* Carruth. from the Paleocene of England can be undoubtedly assigned to *Osmunda* subgenus *Plenasium*. Chandler’s rationale has since served as a precedence for subsequent authors to place other Paleogene, Neogene, and—more recently—also Triassic to Cretaceous fossils of Osmundaceae in genera originally defined for extant species [29, 30, 63, 65, 66, 101–104]. Finally, well-preserved permineralized rhizomes from the Upper Cretaceous of Canada that are strikingly similar to those of modern *Osmunda cinnamomea* have led the authors to even identify a particular extant species in the Mesozoic fossil record [64]. These assignments and new combinations have been adopted in every subsequent systematic treatment of fossil Osmundaceae [7, 36, 37]. Therefore, the identification of extant genera and species of Osmundaceae even in the Mesozoic fossil record is a universally accepted practice, providing the fossils show sufficient diagnostic detail to warrant affiliation with their extant relatives. Fossils that have structural features unknown among modern taxa are, by contrast, usually placed in more or less narrowly defined fossil taxa, such as *Palaeosmunda* R.E.Gould, *Osmundacaulis* C.N.Mill. emend. Tidwell, or *Aurealcaulis* Tidwell & L.R.Parker [7, 36, 71]. The remaining osmundoid fossil rhizomes that cannot be positively assigned to any of these natural groups continue to be placed in the rather broadly defined fossil taxon *Millerocaulis* Tidwell emend. E.I.Vera (including the formerly separated *Millerocaulis* Tidwell emend. Tidwell and *Ashicaulis* Tidwell) [7, 36, 37, 105].

The calcified osmundaceous rhizome described here contains all anatomical features diagnostic of *Osmunda* [11, 30]: (1) ectophloic-dictyoxylic siphonostele with complete leaf gaps; (2) thin parenchymatic inner cortex and distinctly thicker, homogeneous, fibrous outer cortex; (3) heterogeneous sclerenchyma cylinders in the petiole bases; and (4) sclerenchyma fibres in the stipular wings of the petiole. It shares an ample number of characters with subgenera *Osmundastrum* and *Osmunda sensu* Miller, but is markedly distinct from subgenus *Plenasium* [29, 30]. The rather high degree of stele dissection and the distant point of initial bifurcation of leaf-trace protoxylem are typical of *Osmundastrum* and *O. claytoniana* [29, 30]; finally, the presence of usually a single root per leaf trace together with the development of (ultimately) one abaxial arch and two lateral masses of thick-walled fibres in the petiole sclerenchyma ring render the new species particularly similar to subgenus *Osmundastrum* [29, 30]. Since the fossil differs from extant species merely in specific diagnostic characters, we have no hesitation in assigning it to *Osmunda* in accordance with conventional practice [29, 30, 36, 63, 75, 101].

By analogy, the same basic similarity also applies to at least five of the >25 fossil species currently included in *Millerocaulis sensu* Vera and *Ashicaulis*, which are all characterized by having heterogeneous sclerenchyma rings in the petioles: *M. liaoningensis* [106], *A. claytoniites* [107], *A. plumites* [108], and *A. wangii* [109]—all from the Jurassic of China—and *M. johnstonii* from Tasmania [110], which we, therefore, included in our phylogenetic analyses. The holotype of the last of these species was collected from a gravel pit; following Tidwell *et al.* [110], we consider the age of this specimen to be likely concordant with those of other Mesozoic permineralized fern stems from eastern Tasmania, which have recently been dated as Early Jurassic [111].

### Systematic placement of fossil *Osmunda* rhizomes among modern Osmundaceae

#### Phylogenetic network analysis

Relationships among extant species in the distance network based on our morphological matrix are congruent with those of molecular phylogenetic analyses [31, 32], confirming that the morphological matrix based on rhizome anatomy serves well in resolving systematic relationships among modern Osmundaceae. The only major exception is expressed by *O. claytoniana*, which, together with extant species of subgenus *Osmunda sensu* Yatabe *et al.* and Paleogene and Neogene fossils, forms a group essentially consistent with subgenus *Osmunda sensu* Miller*.*

The Jurassic taxa included in our analysis, including *O. pulchella*, form a broad box-like structure that bridges the gap between the relatively derived *Osmundastrum* and the less derived *Osmunda* subgenus *Osmunda sensu* Miller (Fig. 8). Their long terminal branches are due to unique trait combinations intermediate between their more derived fossil and extant relatives. Collectively, the Jurassic species probably represent ancestral forms of *Osmunda s.l.*, some being more similar to *O. cinnamomea* (*O. pulchella*) and others to subgenus *Osmunda sensu* Miller (e.g. *A. wangii*).

Overall, the placement of the other fossil taxa accords well with the basic assumption that they should be less derived—and thus placed closer to the centre of the network—than their extant relatives. However, there is one major exception: *O. dowkeri* from the Paleogene is the furthest-divergent (i.e. most derived) of all fossil *and* extant species in the *Plenasium* group. This relates to its unusually complex stele organization, which is highly dissected and contains by far the largest number of xylem segments of all species analysed (exceeding 30, compared to less than 12 in all other *Plenasium* and less than 20 in most other *Osmunda*).

Notably, a subdivision into two putatively monophyletic subgenera *Osmunda sensu* Yatabe *et al.* and *Claytosmunda* generates two taxa without discriminating anatomical and morphological features (potential aut- or synapomorphies according to Hennig [112]). Miller’s paraphyletic subgenus *Osmunda* accommodates the fossil taxa, whereas the concept of *Osmunda* proposed by Yatabe *et al.* [31, 45] precludes infrageneric classification of most fossil species (Fig. 8).

#### Compatibility with vegetative morphology

The systematic relationships revealed from our analysis of anatomical characters of the rhizomes reflect the distribution of gross morphological and fertile features within Osmundaceae very well. The isolated position and tight clustering of subgenus *Plenasium*, for instance, finds support through morphological data in the form of its invariant, unique frond morphology: unlike any other modern Osmundaceae, all extant *Plenasium* species are characterized by having invariably simple-pinnate and hemi-dimorphic fronds. The rather wide dispersion of the (paraphyletic) subgenus *Osmunda* Miller is congruent with the variable frond morphology and dimorphism in this group, ranging from pinnate–pinnatifid [e.g. *O. claytoniana* (similar to *O. cinnamomea*)] to fully bipinnate and from fully to variably hemi-dimorphic.

The only major topology where anatomical data alone probably fail to generate a realistic divergence distance occurs in the branch including *Todea* and *Leptopteris*. These genera, having a rhizome structure broadly similar to that of *Osmunda* and especially *Osmundastrum* [11] (but see Fig. 8), are characterized by unique vegetative and fertile characters (e.g. isomorphic fronds; tripinnate fronds, arborescent habit, and lack of stomata in *Leptopteris*) that differentiate them very clearly from *Osmunda s.l.*

#### Integrating fossil species into the molecular backbone topology

Overall, the results of the EPA provide good support regarding the relationships between fossil and extant taxa (compare Figs. 8 and 9). However, notable ‘position swaps’ occur between the placements obtained from different weighting methods of several taxa, including *Osmunda pulchella*. This incongruence is due to intermediate character combinations inherent to ancestral taxa, which we interpret to result in ‘least conflicting’ placements at varying root positions; the EPA is designed to optimize the position of a query taxon within a pre-defined backbone topology. Because *O. pulchella* and other fossil taxa have character combinations of genetically distant taxa, the model-based weights in particular will down-weigh the relevant characters. Maximum parsimony has a much more naïve approach in this respect, which may help achieve a more plausible placement of the fossils. Nevertheless, the fact that this down-weighting results in a placement close to the roots, but not in the tips of sub-trees, indicates that the remaining character suite is plesiomorphic in general, thus supporting the interpretation of fossil taxa such as *O. pulchella* as ancestors of extant clades and possibly individual species (Figs. 8 and 9).

#### Summary

Altogether, the results detailed above lead us to the following conclusions about the systematic and phylogenetic placements of fossil species among modern Osmundaceae:

1. The Jurassic *Osmunda pulchella* is an ancestral member of *Osmunda s.l.* combining diagnostic features both of *Osmunda s.str.* and of *Osmundastrum*.
2. Other species reported from the Jurassic, together with *O. pluma* (Paleogene) and *O. wehrii* (Neogene), are representatives of the (paraphyletic) subgenus *Osmunda sensu* Miller, including potential ancestors of extant species of subgenus *Osmunda* and *Claytosmunda.*
3. *Osmunda oregonensis* (Paleogene) is closely allied with subgenus *Osmunda sensu* Yatabe *et al.* (see [30]).
4. *Osmunda arnoldii* and *O. dowkeri* belong to subgenus *Plenasium* and are closely similar to *O. banksiifolia*; the highly derived *O. dowkeri* represents the highest degree of specialization in the subgenus, which is supposed to have reached its heyday in distribution and diversity during the Paleogene [30].
5. A close systematic relationship of extant and all fossil *Osmundastrum* is unambiguous, despite their wide stratigraphic age-span (Cretaceous, Paleogene, and Neogene) and ‘trans-Pacific’ geographic distribution. It is interesting to note, however, that the rhizomes of *O. cinnamomea* show a far greater disparity in anatomical characters than all other subgenera and even genera of modern Osmundaceae, indicating the existence of probably more than just a single *Osmundastrum* species in the past (Fig. 8).
6. *Osmunda iliaensis* and *O. shimokawaensis* are most likely representatives of that species complex of subgenus *Osmunda* that is today restricted to East Asia (i.e. *O. lancea* and *O. japonica*); *O. shimokawaensis* may be ancestral to *O. japonica* and *O. lancea*.
7. the Early Cretaceous *Todea tidwellii* may be as related to modern *Leptopteris* as it is to *Todea*.

### Re-evaluation of the Generic Status of *Osmundastrum*

The intermediate character combination and the resulting systematic placement of *Osmunda pulchella* and other Jurassic species between *Osmundastrum* and subgenus *Osmunda* Miller challenges the current treatment of *Osmundastrum* as a separate genus. In the following section, therefore, we provide a detailed re-evaluation of the sum of evidence that has been used to invoke generic separation of *Osmundastrum*. We begin with what is perhaps considered the most novel and reliable body of evidence—molecular data—and continue with additional evidence from morphological, anatomical, and hybridization studies.

#### Molecular data

The comprehensive multi-locus phylogeny of Metzgar *et al.* [32] has recently been interpreted to fully support a separate generic status of *Osmundastrum* as suggested earlier by Yatabe *et al.* [31]. Inter-generic and inter-subgeneric ingroup-only relationships based on the molecular matrix employed by Metzgar *et al.* (reproduced here in Fig. 10) indeed receive nearly unambiguous support from the concatenated gene matrix.

Our analysis of the root-placement stability, however, revealed that the paraphyletic status of *Osmunda s.l.* inferred from the results of Metzgar et al. [32] is not unambiguously supported by all gene regions (Fig. 10). Whereas this scenario indeed receives strong support from the two coding regions (*rbc*L-gene, *atp*A-gene), the molecular data matrix also yields a strong conflicting signal from three relatively conserved spacer sequences (i.e. *atp*B-*rbc*L, *rbc*L-*acc*D, and *trn*L-*trn*F) that indicates an alternative root placement between *Leptopteris*–*Todea* and the remaining *Osmunda s.l.* This latter signal offers an equally valid interpretation that would resolve *Osmunda s.l.* as monophyletic.

The root-placement problem may be due in part to the insufficiently comprehensive selection of out-group taxa, which is limited to four samples of leptosporangiate ferns in the matrix of Metzgar *et al.* [32]: *Matonia pectinata* R.Br. (Matoniaceae), *Dipteris conjugata* Reinw. (Dipteridaceae) and *Gleicheniella pectinata* (Willd.) Ching and *Diplopterygium bancroftii* (Hook.) A.R.Sm. (Gleicheniaceae)—all members of Gleicheniales. Current fern phylogenies indicate that Osmundaceae represent the earliest-diverged group in the Polypodiopsida, which include five other extant orders apart from Gleicheniales (see e.g. [1, 2, 4]). We anticipate that a less ambiguous molecular signal may be obtained by the selection of a more comprehensive range of outgroup taxa, including representatives from all major lineages within the Polypodiopsida (in particular Hymenophyllales and Schizaeales) and the sister clades of this class (Equisetopsida and Marattiopsida). Comprehensive sampling of slowly evolving nuclear genes (see e.g. [67, 68]) for the ingroup and outgroup may help to identify outgroup-inflicted branching artefacts in the current plastid-sequence-based topology. Because representatives of Gleicheniales are relatively derived in comparison to Osmundales, they may inflict outgroup long-branch attraction with *Osmundastrum* [see Additional file 2: Figure. S1 (note the long terminal edge bundles) and S2 in ESA].

#### Anatomy

Rhizomes of extant *O. cinnamomea* have several peculiar and supposedly unique characters, including (1) the common occurrence of an internal endodermis; (2) the rare occurrence of a dissected, ectophloic to amphiphloic stele; (3) bifurcation of the protoxylem bundle only as the leaf trace enters the petiole base; (4) the sclerenchyma ring of a petiole base containing one abaxial and two lateral masses of thick-walled fibres; (5) usually single, rarely paired roots arising from the leaf traces; and (6) a patch of sclerenchyma adaxial to each leaf trace in the inner cortex (e.g., [11, 30]).

The first two characters occur inconsistently in extant individuals, and are notably absent in fossil (Cretaceous to Neogene) representatives of *Osmundastrum* [29, 30, 64], suggesting that these might represent recently acquired traits [30]. Moreover, dissected steles and dictyosteles, with either two endoderms or two phloem layers connecting through a leaf gap, are conditions only rarely and inconsistently developed below incipient rhizome bifurcations [8, 9, 11]. The significance of both characters as diagnostic features of *Osmundastrum* is thus questionable.

The point of protoxylem bifurcation and the distribution of patches of thick-walled fibres in the petiole sclerenchyma ring are consistent and arguably appropriate diagnostic characters of *Osmundastrum*. However, among the remaining *Osmunda s.l.* species, these same characters are regarded as diagnostic only at specific or subgeneric rank [30]. Thus, it would seem inconsistent to afford greater taxonomic weight to these characters in the delimitation of *Osmundastrum* alone.

Roots typically arising singly is a useful character discriminating *Osmundastrum* and *Osmunda pulchella* from the remaining *Osmunda*, although this feature is inconsistent and may be difficult to observe [11, 30]. The occurrence of sclerenchyma patches adaxial to the leaf traces in the inner stem cortex is the only invariant and unique character of *Osmundastrum* that we consider might validate its separation beyond species level. Apart from *Osmundastrum*, this feature occurs also in *Todea* but not in its sister genus *Leptopteris* [30].

#### Morphology

Morphological features commonly regarded as diagnostic of *Osmundastrum* include (1) generally complete frond dimorphism; (2) pinnate–pinnatifid frond architecture; and (3) dense abaxial trichomes on pinna rachides [32]. However, using frond architecture and dimorphism as a strict diagnostic character has been shown to be problematic (e.g. [11]). Pinnate fronds with deeply pinnatifid segments occur in both *O.* (*Osmundastrum*) *cinnamomea* and *O.* (*Claytosmunda*) *claytoniana*. Moreover, some common varieties and growth forms of *O. cinnamomea* produce only hemi-dimorphic fronds [113–116], some having apical fertile portions resembling those of *O. regalis* (see, e.g. [114, 117, 118]) and others having intermittent fertile portions like those of *O. claytoniana* (see, e.g. [114, 119]). Further, completely dimorphic fronds are also predominant in *O. lancea*, common in *O. japonica*, and sporadic in *O. regalis* ([11, 120]). Significantly, such ranges of variation are encountered only in the species complex including *Osmundastrum* and *Osmunda* subgenus *Osmunda* Miller (= subgenera *Claytosmunda* and *Osmunda* Yatabe *et al.*).

Finally, fronds of all *Osmunda s.l.* species emerge with a more-or-less dense abaxial indumentum and differ merely in the duration to which the trichome cover is retained in the course of frond maturation [11]. In fully mature fronds of all species considered, most of the hair cover is ultimately lost, with *O. cinnamomea* [especially *O. cinnamomea* var. *glandulosa* Waters [121, 122] merely tending to retain greater amounts of hairs than *O. claytoniana*, and those in turn more than other species [11]. In summary, we follow Hewitson [11] in arguing that none of these morphological features provide consistent and reliable diagnostic characters for separating *Osmundastrum* from subgenus *Osmunda* Miller.

#### Hybridization

Metzgar *et al.* ([32] p. 34) suggested that the existence of hybrids can be used to decide about the elevation of subgenera to generic ranks. Numerous natural hybrids, intra- and inter-subgeneric, are known to occur in *Osmunda s.str.*: *O.* × *ruggii* R.M.Tryon in eastern North America (*O. regalis* × *O. claytoniana*; [49, 51]), *O.* × *mildei* C.Chr. in southern China (*O. japonica* × *O. vachellii* Hook.; [123, 124]), *O*. × *hybrida* Tsutsumi, S.Matsumoto, Y.Yatabe, Y.Hiray. & M.Kato in Southeast Asia (*O. regalis* × *O. japonica*; [68]), and *O*. × *intermedia* (Honda) Sugim. (*O. japonica* × *O. lancea*) and *O.* × *nipponica* Makino (*O. japonica* × ?*O. claytoniana*) in Japan [23, 67, 124]. The apparent absence of naturally occurring hybrids involving *Osmundastrum* has been interpreted to result from its particularly isolated position within *Osmunda s.l.* [29, 30]. However, Klekowski [50] conducted artificial breeding experiments and readily succeeded in producing viable hybrid sporophytes from *O. cinnamomea* × *O. claytoniana* and *O. cinnamomea* × *O. regalis*, with equal or even higher yields (1 out of 8 and 2 out of 9, respectively) compared to *O. claytoniana* × *O. regalis* (1 out of 8). In addition, some authors suspect that there may also be natural hybrids between *O. cinnamomea* and *Osmunda s. str.* (see [67]). So far, there is no record of hybridisation between *Leptopteris-Todea* and *Osmunda s.l.* either *ex situ* or *in situ* (e.g., from southern Africa, where the geographic ranges of *Osmunda* and *Todea* overlap [125]).

#### Summary

We find that neither molecular, anatomical, morphological, nor hybridization studies have yet succeeded in providing unequivocal evidence that would warrant separate generic status of *O. cinnamomea,* reject an (inclusive) common origin of *Osmundastrum* and *Osmunda s.str*., or else identify an (inclusive) common origin of *Leptopteris*-*Todea* and *Osmunda s.str*. Rather we argue that the sum of evidence for extant taxa detailed above allows for two equally valid hypotheses: the ‘paraphyletic-*Osmunda* scenario’ [31, 32] and an alternative ‘monophyletic-*Osmunda* scenario’ [30]*.*

### The impact of *Osmunda pulchella* on the classification of modern Osmundaceae

**Fig. 12:**
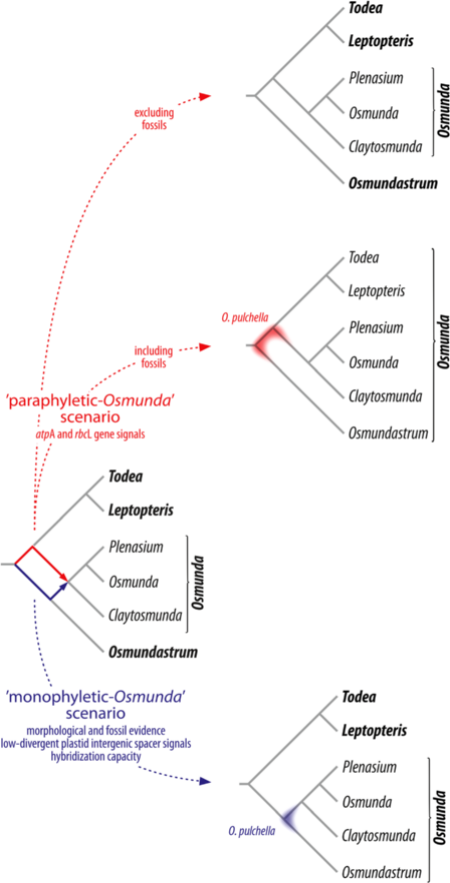
Diagram illustrating the critical significance of the placement of *Osmunda pulchella* for a strictly cladistic-systematic classification of modern Osmundaceae regarding the two alternative rooting schemes (Figs. 10, 11). Genera names in bold; infrageneric taxa names in regular font

The phylogenetic placement of *Osmunda pulchella* is critical to the systematic classification of modern Osmundaceae (Figs. 11, 12). In the specified topology of the ‘paraphyletic *Osmunda* scenario’, most parsimonious placement of *O. pulchella* is at the base of the tree, at the root of either *Osmundastrum* or of the remaining *Todea*-*Leptopteris-Osmunda s.str.* clade (Fig. 11). If this phylogenetic scenario is followed, and if only holophyletic groups are considered valid taxonomic units (see, e.g., [126, 127] for critical discussion), then it follows that all modern Osmundaceae need be included in one genus *Osmunda*, with *Plenasium*, *Osmunda*, *Claytosmunda*, *Osmundastrum*, *Todea*, and *Leptopteris* being infrageneric taxa (Fig. 12). Alternatively, the ‘four-genus classification’ proposed by Yatabe *et al.* and Metzgar *et al.* could of course also be maintained under the ‘paraphyletic *Osmunda* scenario’ if fossil taxa were to be excluded from systematic classification as a whole (Fig. 12). We expect, however, that such practice would be broadly met with criticism from palaeobiologists and neontologists (see e.g. [59, 60, 69, 70]); in the present study, it would be particularly ignorant not to place the new fossil in a systematic context given that it fully agrees with the circumscription of an extant genus that is diagnosed by a considerable number of informative anatomical characters.

If, by contrast, the specified topology of the ‘monophyletic *Osmunda* scenario’ is followed, in which the most parsimonious placement of *O. pulchella* is as sister to *O. cinnamomea* at the base of an *Osmundastrum-Osmunda s.str.* clade (Fig. 11), then all fossil and extant species of modern Osmundaceae can be resolved in three mutually monophyletic genera: *Todea*, *Leptopteris*, and *Osmunda*, the last of these including the subgenera *Plenasium*, *Osmunda*, *Claytosmunda*, and *Osmundastrum* (Fig. 12).

In our opinion, this latter option integrates the apparently conflicting evidence from studies of the morphology, anatomy, molecular data, and fossil record of Osmundaceae in a much more realistic and elegant way, and offers a more practical taxonomic solution. We, therefore, argue that *Osmunda pulchella* described here exposes the recently established paraphyly of *Osmunda s.l.* as a result of a sampling or reconstruction artefact in the molecular matrix employed. A broader outgroup selection and more comprehensive gene sampling (e.g. including nuclear genes) may resolve the root of Osmundaceae more reliably in the future, providing a molecular data set can be assembled that is immune to outgroup long-branch attraction.

### Evolutionary significance of fossil *Osmunda* rhizomes

**Fig. 13:**
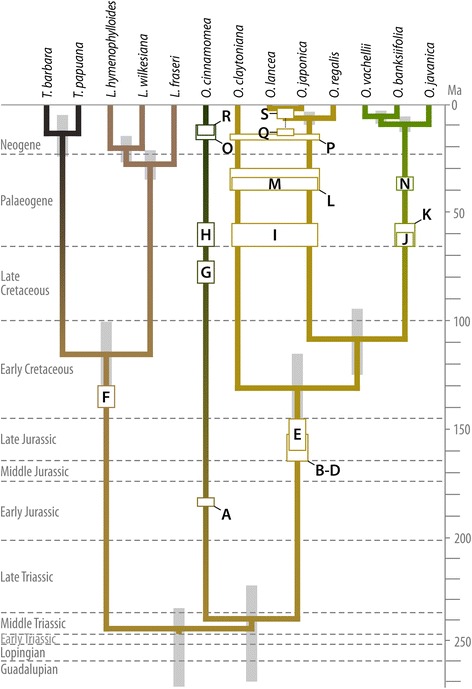
Diagram illustrating the phylogenetic positions of fossil rhizomes within an independently obtained chronogram for modern Osmundaceae that was calibrated using frond fossils only [34]. **a** = *O. pulchella*; **b**–**d** = *M. liaoningensis*, *A. plumites*, and *A. wangii*; **e** = *A. claytoniites*; **f** = *T. tidwellii*; **g** = *O. cinnamomea* (Cretaceous, Canada [64]); **h** = *O. precinnamomea* [29]; **i** = *O. pluma*; **j** = *O. dowkeri* (Paleocene, UK [75]); **k** = *O. arnoldii*; **l** = *O. oregonensis*; **m** = *O. nathorstii*; **n** = *O. dowkeri* (Eocene, USA; see [29]); **o** = *O. cinnamomea* (Neogene, USA [29]); **p** = *O. wehrii*; **q** = *O. shimokawaensis*; **r** = *O. cinnamomea* (Miocene, Japan [63]); **s** = *O. iliaensis*

Grimm *et al.* [34] recently used the rhizome fossils and molecular data studied herein together with an additional set of 17 frond fossils to infer divergence ages for the major splits within modern Osmundaceae. Among several tests, the authors employed a ‘fossilized-birth-death’ (FBD) Bayesian dating approach in which only the frond fossils were used for the calibration of age-distribution priors. The results of this test provide an independent temporal framework [34: supplement] that can be used to assess the evolutionary significance of fossil *Osmunda* rhizomes (Fig. 13).

Calibrated using only frond fossils, the FBD approach dated the split between *Osmundastrum* and the remaining *Osmunda* as being older than mid-Late Triassic. Consequently, Jurassic rhizomes with intermediate or plesiomorphic anatomy represent either precursors or extinct sister lineages of extant clades within *Osmunda s.l*. With its Early Jurassic age and its intermediate anatomical character suite, *O. pulchella* emerges as an ideal candidate for a true precursor of subgenus *Osmundastrum* (Fig. 13a), which became established in its present form by the Late Cretaceous (Fig. 13g). The other Jurassic rhizomes have plesiomorphic character suites shared with all remaining *Osmunda*, but lack the apomorphic states that are characteristic of the highly specialized subgenus *Plenasium* (Fig. 13b–e). This is in consonance with the mid-Cretaceous root age inferred for *Plenasium*, which predates the occurrence of the oldest known *Plenasium* rhizome fossils by at least 30 million years (Fig. 13j, k). In most other cases, the estimated divergence ages also predate the earliest rhizome fossils with lineage-specific characters, as would be expected. One conflict occurs in the seemingly precocious appearance of *O. shimokawaensis* (Fig. 13q), which our analyses identify as a precursor of the two East Asian *Osmunda* species, in the late middle Miocene (12–14 million years ago). According to the FBD dating calibrated via frond fossils, the split between these two species (*O. lancea* and *O. japonica*) and *O. regalis* occurred less than 10 million years ago. However, this conflict can be explained by the species-level molecular data used in the dating, which mask the substantial intraspecific genetic disparity between New-World and Old-World populations of *O. regalis* [68].

Finally, it needs to be pointed out that—following the evidence gathered and presented here—Grimm *et al.* [34] did not employ both rooting scenarios in their dating. However, during earlier stages of that study, preliminary dating analyses were performed for each of the two different rooting scenarios, using a dirichlet probability prior (DPP) model and including oldest fossils as minimum age constraints for the hypothetical most-recent common ancestors of extant taxa (Additional file 3). Dating using the DPP model shares the basic principles of FBD dating, except that fossils are used in the traditional way as node-height constraints. The results showed that the choice of the rooting scenario is largely irrelevant to the estimated subsequent divergence ages. Thus, even if future studies should produce more comprehensive and better-substantiated evidence in favour of a paraphyletic rooting scenario over the monophyletic scenario, *O. pulchella* would still remain a likely member of the *Osmundastrum* lineage.

## Conclusions

(i) *Osmunda pulchella* sp. nov. from the Early Jurassic of Sweden is among the earliest unequivocal records of fossil *Osmunda* rhizomes, and a likely precursor of the extant *O. cinnamomea* and its fossil relatives.
(ii) Intermediate anatomical character suites of *O. pulchella* and other Jurassic osmundoid rhizomes support re-inclusion of the recently separated, monospecific *Osmundastrum* within *Osmunda*.
(iii) The sum of morphological, anatomical, molecular, and fossil evidence supports modern *Osmunda* (including *Osmundastrum*) and *Todea-Leptopteris* being mutually monophyletic.
(iv) The recently established rooting of Osmundaceae and the resulting paraphyly of *Osmunda s.l.*, based solely on molecular data, likely results from a sampling or reconstruction artefact. More comprehensive outgroup selection and broader gene sampling may hold the potential to alleviate this problem in future analyses.
(v) Our results accord remarkably well with independently obtained divergence ages based on molecular dating calibrated via frond fossils.

## Methods

### Fossil material

The studied specimen was collected from mafic volcaniclastic deposits (“Djupadal formation” [61]) near Korsaröd lake (Höör municipality, central Skåne, Sweden). The host strata are interpreted to be local remnants of ash falls and lahar flows that spread from a nearby volcanic centre, similar to other occurrences of mafic volcaniclastic and epiclastic deposits associated with basaltic necks in central Skåne [128]. Palynological analyses indicate a late Pliensbachian (late Early Jurassic) age [62], which agrees well with radiometric dating of associated basaltic necks that place the peak phase of volcanism in central Skåne in the Pliensbachian to Toarcian (*ca* 183 Ma [129]). Petrographic thin sections (Figs. 1, 2, 3, 4, and 5) were studied and photographed using an Olympus BX51 compound microscope with an attached Olympus DP71 digital camera. Two sectioned blocks of the holotype were selected for SEM analyses; the sectioned surfaces of these blocks were polished, etched with 5 % HCl for 5–10 s, mounted on aluminium stubs, coated with gold for 90 s, and finally analysed using a Hitachi S-4300 field emission scanning electron microscope at the Swedish Museum of Natural History (Fig. 6). We applied conventional adjustments of brightness, contrast, and saturation to most of the digital images using Adobe® Photoshop® CS5 Extended version 12.0; in some cases, we performed manual image stitching and image stacking [130] in order to obtain sufficiently sharp, large composite images with optimal depth of field.

### Phylogenetic analyses

In order to place the newly described fossil in a phylogenetic context, we assembled a morphological matrix that is based on the phylogenetic assessment of Miller [30], including all extant and fossil members of the extant genera plus those fossil rhizome species that agree in all anatomical features with those of modern genera [see Additional file 1; ESA].

#### Network analysis

We rely exclusively on network methods as implemented in SplitsTree v. 4.13.1 [131] to draw phylogenetic conclusions based on the morphological matrix (see [58, 132–135]): (1) a neighbour-net [136, 137] based on mean inter-taxon distances, and (2) bipartition networks to visualize support (Bayesian-inferred posterior probabilities, PP; non-parametric bootstrapping, BS) for alternative phylogenetic relationships [58, 138, 139]. BS support was established under three commonly used optimality criteria using 10,000 bootstrap replicates: (1) Least-squares via the BioNJ algorithm (BS_NJ_; [140]); (2) Maximum parsimony (BS_MP_) using PAUP* [141, 142]; and (3) Maximum likelihood (BS_ML_) via the fast bootstrapping implementation in RAxML v. 7.4.2 [143, 144] using both available transition models for categorical (multistate) data, i.e. (*i*) the general time-reversible model (BS_ML/GTR_) [145] and (*ii*) Lewis’ model (BS_ML/MK_) [146]. For configuration details of Bayesian inference, non-parametric bootstrapping, and network-wise visualization refer to Additional file 1 [ESA].

#### Re-visiting the Osmundaceae root

We analysed the root placement in the phylogenetic tree of Metzgar *et al*. [32] using the original molecular matrix. First, a set of traditional phylogenetic analyses was run, including a gene jackknifing procedure. Trees and bootstrap support were inferred using the concatenated data, each gene partition separately, and matrices in which one partition was deleted. Second, the evolutionary placement algorithm (EPA [147, 148]) as implemented in RAxML was used to determine the optimal position of the outgroup taxa (i.e. the position of an outgroup-inferred root) within an ingroup-only topology. The EPA has been originally designed for placing fossils [147] or short-sequence reads [148], but its metrics can also be used to generally test the position of one or many query sequences—here: outgroup taxa—in a given topology—here: an ingroup-only ML tree—in a ML framework (A. Stamatakis, pers. comm., 2014).

#### Character plotting and independent optimization of the placement of fossils within a molecular framework of modern taxa

Using the EPA we estimated a weight (probability) for the placement of our fossil within the molecular backbone topology reproduced from the data matrix of Metzgar *et al.* [32]. We also determined the most parsimonious placement of the newly described fossil within the molecular tree of Metzgar *et al.* [32] implemented into the morphological matrix using Mesquite v. 2.75 [149]; this is done by simply moving the fossil within the given topology and recording the incremental increase in steps added to the resulting whole tree-length (see [133, 150] for applications).

### Nomenclatural remark

In order to maintain consistent use of terminology, we employ the following names: (1) *Osmunda cinnamomea* instead of the currently used *Osmundastrum cinnamomeum* (L.) C.Presl; (2) ‘modern Osmundaceae’, referring to those genera of Osmundaceae that are based on extant species, i.e. *Osmunda* (including *Osmundastrum*), *Todea*, and *Leptopteris*; (3) ‘*Osmunda s.l.*’, referring to the traditional generic concept that includes all extant and several fossil species (e.g., Miller, 1971); and (4) ‘*Osmunda s.str.*’, referring to the recently proposed generic concept of *Osmunda* that excludes *O. cinnamomea* and *O. precinnamomea* C.N.Mill. (i.e., including only *Osmunda* subgenera *Osmunda, Claytosmunda* and *Plenasium*) [31]. Where necessary, we cite taxon authorities to discriminate between formal subgeneric concepts used by Miller [30] and Yatabe *et al.* [45].

For naming the additional Jurassic species included in our analysis, we follow Vera’s [99] taxonomic revision of *Millerocaulis* and *Ashicaulis*, in which a more broadly defined generic concept of *Millerocaulis* was proposed to include also those species that were previously assigned to *Ashicaulis* [99]. Vera’s concept, however, has so far not been universally adopted, and the genus *Ashicaulis* is still frequently used; new *Ashicaulis* species that have been introduced since the revision appeared [101–103] are provisionally listed here with their original names and an additional remark; these include *Ashicaulis* (=*Millerocaulis sensu* Vera [99]) *claytoniites* [101], *A.* (=*Millerocaulis sensu* Vera [99]) *plumites* [102], and *A.* (=*Millerocaulis sensu* Vera [99]) *wangii* [103].

### Availability of supporting data

An electronic supplementary data archive (ESA) containing all original data files and results, including the employed matrices in NEXUS format is available for anonymous download at www.palaeogrimm.org/data/Bfr15_ESA.zip [please refer to the accompanying index document (GuideToFiles.txt) for a detailed description].

## Additional files

Additional file 1:
**Document detailing definition and coding of characters in the morphological matrix, and the set-up for phylogenetic analyses and the evolutionary placement algorithm.**
Additional file 2:
**Neighbour net inferred from uncorrected pairwise distances based on the concatenated data set of Metzgar et al. (2008).** Bootstrap support of (alternative) splits is annotated. Note the occurrence of four genetically distinct lineages; the splits that place *O. cinnamomea* as sister to all other Osmundaceae received strong support only from atpA and rbcL partitions. Additional file 3:
**Divergence ages and 95 % height posterior densities obtained with fossilized birth-death dating [** 34 **] and the DPP model using five minimum age constraints for calibration (P. Kapli, G. Grimm, unpubl. data).**
