# Supplementary material for: Osmunda pulchella sp. nov. from the Jurassic of Sweden—reconciling molecular and fossil evidence in the phylogeny of modern royal ferns (Osmundaceae)
Source: BMC Evol Biol. 2015 Jun 30;15:126. doi: 10.1186/s12862-015-0400-7 (PMC4487210; doi:10.1186/s12862-015-0400-7)
Supplement: Additional file 1: — Document detailing definition and coding of characters in the morphological matrix, and the set-up for phylogenetic analyses and the evolutionary placement algorithm. [file 12862_2015_400_MOESM1_ESM.pdf]

Osmunda pulchella sp. nov. from the Jurassic of Sweden—reconciling molecular and fossil evidence in the phylogeny of modern royal ferns (Osmundaceae)

Benjamin Bomfleur, Guido W. Grimm, and Stephen McLoughlin
Swedish Museum of Natural History, Department of Palaeobiology, Svante Arrhenius Väg 7, SE-10405 Stockholm, Sweden

SUPPLEMENTARY FILE S1. DETAILED METHODS

Definition and coding of characters in the morphological matrix

Remarks. — Characters 1–5, 12, 15, 16, 25, and 31 have been excluded from the analyses since they are either invariant within the current taxon set or not applicable to fossil taxa; *Todea papuana*, *Osmunda kidstonii*, and *O. nathorstii* included in the matrix were not considered in the analyses of the morphological matrix due to missing data.

Character 1 [excluded]. — Petiole bases (character I.C of Miller, 1971): (0) closely adhering; (1) loosely adhering.

Character 2 [excluded]. — Blade architecture (character III.O of Miller, 1971): (0) pinnate; (1) bipinnate; (3) bipinnate-pinnatifid or tripinnate.

Character 3 [excluded]. — Fertile frond dimorphism (character III.Q of Miller, 1971): (0) absent; (1) modified pinnae on incompletely dimorphic fronds; (2) fully dimorphic fertile fronds.

Character 4 [excluded]. — Stele type (modified from character I.A of Miller, 1971): (0) protostele; (1) siphonostele; (2) dictyostele.

Miller used this character to differentiate taxa with “ultraspecialized” steles (Miller, 1971: p. 152); these, however, can alternatively be delimited using characters defining phloem and endodermis positions. All “modern Osmundaceae” (see nomenclatural remark in main text: material and methods) are characterized by simple siphonosteles.

Character 5 [excluded]. — Composition of central portion of protostele (separated and modified from character I.A of Miller, 1971; see comment above): (0) long tracheids; (1) short tracheids; (2) tracheids and small amounts of parenchyma.

Character 6. — Phloem position (character II.D of Miller, 1971): (0) external only; (1) external and (rarely) internal.

Character 7. — Endodermis (character II.E of Miller, 1971): (0) external only; (1) external and rarely internal; (2) external, internal, and rarely connecting through leaf gaps.

Character 8. — Sclerenchyma in pith (character II.B of Miller, 1971): (0) absent; (1) present, scattered; (2) variable to completely sclerified.

Character 9. — Xylem cylinder thickness (character II.C of Miller, 1971): (0) 5–15 cells thick; (1); 20–30 cells thick; (2) > 50 cells thick.

Character 10. — Degree of stele dissection as expressed in the number of xylem segments per mm stele perimeter in transverse section (modified from character III.R of Miller, 1971): (0) < 1.3; (1) [1.4;1.9]; (2) > 2.1.

Miller (1971) recognized an evolutionary trend in the segmentation of the stele in fossil and modern *Osmunda,* from many (>40) over medium (20–40) to few segments (<20). Most species covered here would fall in Miller's category "R" (see Miller, 1971: caption to text-fig. 8). Larger steles are likely to show more segments than smaller steles and significant variation can be found within some taxa (e.g. 20–33 in *O. dowkeri*; see File S2). In the case of fossils, thin steles with few segments may simply represent younger individuals or more proximal rhizome parts. To compensate for a mere size and ontogenetic effect, we used the following formula (**Formula 1**):

$$XSpPSt = \frac{N(XS)_{max}}{d(Stele)_{max} * \pi} \quad (\textbf{Formula 1})$$

in which $N(XS)_{max}$ = maximum number of xylem segments; $d(Stele)_{max}$ = maximum stele diameter.

Final values ranged between 0.37 (weakly segmented steles) in the fossil *Todea tidwellii* and 2.84 (highly segmented steles) in the extant *Osmunda vachelii*. We then performed a *k*-clustering with Cluster 3.0 (de Hoon *et al*., 2004) to categorize the obtained quantitative data as binary (k = 2) or ternary (k = 3) characters. The *k*-clustering approach is a simple cluster algorithm which allows dividing any kind of quantitative data into a pre-set amount of groups (clusters). We then chose the *k* value (here: k = 3) that would provide distinct groups and a clear cut-off between scorings (Table S1.1). Following the recommendation in the manual for small data sets with a structure like ours we applied the *k-median* algorithm with 'city-block' distances as similarity metric and 10,000 repetitions.

TAB. S1.1. The range of values and the results of the *k*-clustering for *XSpPSt*.

Category	"0"	"1"	"2"	Minimum difference between categories
k = 2	0.37–1.78	1.87–2.86	n/a	0.09
k = 3	0.37–1.27	1.40–1.91	2.12–2.86	0.13

Character 11. — Number of protoxylem poles per leaf trace (character 16 of Wang *et al.*, 2013): (0) one; (1) two.

Character 12 [excluded]. — Leaf-trace protoxylem (character I.D of Miller, 1971): (0) basally mesarch; (1) basally endarch.

This character is inconsistently used in the literature, since the initiation of leaf traces with otherwise endarch protoxylem position can be considered mesarch *before emission from the stem*. We here follow Miller's original definition and determine protoxylem position *at the point of divergence from the stem*. Following this approach, modern Osmundaceae show a basally endarch leaf-trace protoxylem (Miller, 1971: text-fig. 6).

Character 13. — Number of roots per leaf trace (character III.F of Miller, 1971): (0) one, occasionally two; (1) two, occasionally one.

Character 14. — Level of initial bifurcation of leaf-trace protoxylem (modified from characters I.F and II.E of Miller, 1971): (0) outer cortex to petiole base; (1) in inner, rarely outer cortex.

Miller (1971) put strong emphasis on this character, which he classified into four character states ["in outer cortex to petiole base", "near boundary of inner and outer cortex", "in inner cortex", and "in stele" (the latter being a putative synapomorphy of subgenus *Plenasium*)] weighted with distance values up to 10 in his analysis of all species (matrix I) and into three categories (weighted as 0, 0.5, and 1) in that of the *Osmundacaulis kolbei* line (matrix II). We argue that the character cannot be accurately determined enough to warrant the classification into that many categories; moreover, the scoring of *Plenasium* is problematic, since leaf traces in this subgenus arise from two protoxylem strands that initiate independently in two adjacent stem-xylem segments. Hence, we treat this character as being not applicable in *Plenasium* species (scored as "?").

Character 15 [excluded]. — Composition of cortex (character I.B of Miller, 1971): (0) completely parenchymatic; (1) parenchyma with sclerified outer layer.

Character 16 [excluded]. — Relative breadth of cortical cylinders (character II.F of Miller, 1971): (0) inner layer about as thick as outer; (1) inner layer thinner than outer.

Character 17. — Composition of the inner cortex (character II.G of Miller, 1971): (0) parenchyma only; (1) parenchyma with scattered stone cells; (2) parenchyma with a nest of fibres adaxial to each departing leaf trace.

Character 18. — Number of leaf traces per mm^2 inner cortex in transverse section (modified from character I.E of Miller, 1971): (0) ≤ 0.5; (1) ≥ 0.6.

Analogous to the clustering approach as described in the scoring of character 10, we scored the number of leaf-traces visible in the inner (*NLTIC*, **Formula 2**) and outer cortex (*NLTOC*; see Char. 20, **Formula 3**) divided by area. Following the results of the cluster analysis, the binary scoring provided the most distinct *NLTIC* cut-off values (Table S1.2). The cross-sectioned areas of the inner and outer cortex, respectively, were roughly estimated based on the maximum stele diameter and the thickness of inner cortex (see denominator in **Formula 2**), and additionally the maximum stem diameter (see denominator in **Formula 3**).

$$NLTIC = \frac{N(LT)_{max}}{\pi * \left(\left(\frac{d(stele)_{max}}{2} + t(IC)_{max} \right)^2 - \left(\frac{d(stele)_{max}}{2} \right)^2 \right)} \qquad \textbf{Formula 2}$$

$$NLTOC = \frac{N(LT)_{max}}{\pi * \left(\left(\frac{d(stem)_{max}}{2} \right)^2 - \left(\frac{d(St)_{max}}{2} + t(IC)_{max} \right)^2 \right)} \qquad \textbf{Formula 3}$$

in which $N(LT)_{max}$ = maximum number of leaf-traces in either inner or outer cortex; $d(stele)_{max}$ = maximum stele diameter, $d(stem)_{max}$ = maximum stem diameter; and $t(IC)_{max}$ = maximum thickness of the inner cortex.

TAB. S1.2. The range of values and the results of the *k*-clustering for *NLTIC*.

Category	"0"	"1"	"2"	Minimum difference between categories
k = 2	0–0.51	0.64–2.27	n/a	0.13
k = 3	0–0.28	0.33–0.64	0.74–2.27	0.05

Character 19. — Composition of outer cortex (new character): (0) homogeneous; (1) heterogeneous.

This character is based on observations of Hewitson (1962) and Miller (1967, 1971), and is comparable to character 1243 in the matrix of Jud *et al.* (2008).

Character 20. — Number of leaf traces per mm^2 outer cortex in transverse section (modified from character I.E of Miller, 1971; **Formula 3**): (0) ≤ 0.3; (1) > 0.3.

Following the results of the cluster analysis, the binary scoring provided the most distinct *NLTOC* cut-off values (Table S1.3). See character 18 for details.

TAB. S1.3. The range of values and the results of the *k*-clustering for *NLTOC*.

Category	"0"	"1"	"2"	Minimum difference between categories
k = 2	0.02–0.30	0.38–0.98	n/a	0.08
k = 3	0.02–0.14	0.18–0.28	0.38–0.98	0.04

Character 21. — Sclerenchyma in concavity of petiolar vascular bundle (modified coding of character II.J in Miller, 1971): (0) absent or much reduced; (1) one mass or lining band.

Miller (1971) recognizes seven different states for this character, which we split into three binary characters (characters 21–23) to account for the complex differentiation patterns seen in modern Osmundaceae.

Character 22. — Sclerenchymatic band in concavity of petiolar vascular bundle (modified coding of character II.J in Miller, 1971): (0) simple; (1) bifurcating.

See comment on character 21.

Character 23. — Initiation of sclerenchymatic band in concavity of petiolar vascular bundle (modified coding of character II.J in Miller, 1971): (0) in petiole only; (1) extending downward into stem.

See comment on character 21.

Character 24. — Thickness of sclerenchyma ring (character III.I of Miller, 1971): (0) thicker than vascular strand; (1) thinner than vascular strand.

Character 25 [excluded]. — Sclerenchyma ring of petiole base (modified coding of character II.K of Miller, 1971): (0) homogeneous; (1) heterogeneous, initially forming an abaxial arch of thick-walled fibres.

The development of a heterogeneous sclerenchyma ring in the petiole is considered a critical diagnostic character for the identification of modern Osmundaceae in the fossil record (see Miller, 1971; Rothwell, 2002; Vera, 2008); fossil rhizomes that are superficially similar to those of modern *Osmunda* but that lack the heterogeneous sclerenchyma ring have been initially assigned to *Osmundacaulis* (Miller, 1971) and are currently accommodated in *Millerocaulis* (Tidwell, 1986) or *Ashicaulis* (Tidwell, 1994; see Vera, 2008, for a critical discussion of the genera).

The configuration of patches of particularly thick-walled fibres in the petiole sclerenchyma ring is highly diagnostic (Hewitson, 1962; Miller, 1967, 1971), and is scored in the four following characters.

Character 26. — Abaxial arch differentiating into two lateral masses (modified coding of character II.K of Miller, 1971): (0) no; (1) yes.

Miller used six different states in a single character to account for the complex configuration of the patches of thick-walled fibres (Miller, 1971: text-fig. 7) under the assumption that one type evolved from the other. However, the newly described *Osmunda pulchella* shows that similar patterns can result from different differentiation processes. We accommodated this by translating the development and upward differentiation of these patches into a series of four binary characters (characters 26–29; Fig. S1.1). Alternatively, two ternary characters could be used; this, however, would lead to a lower resolution and less balanced weighting of the various types (Table S1.4).

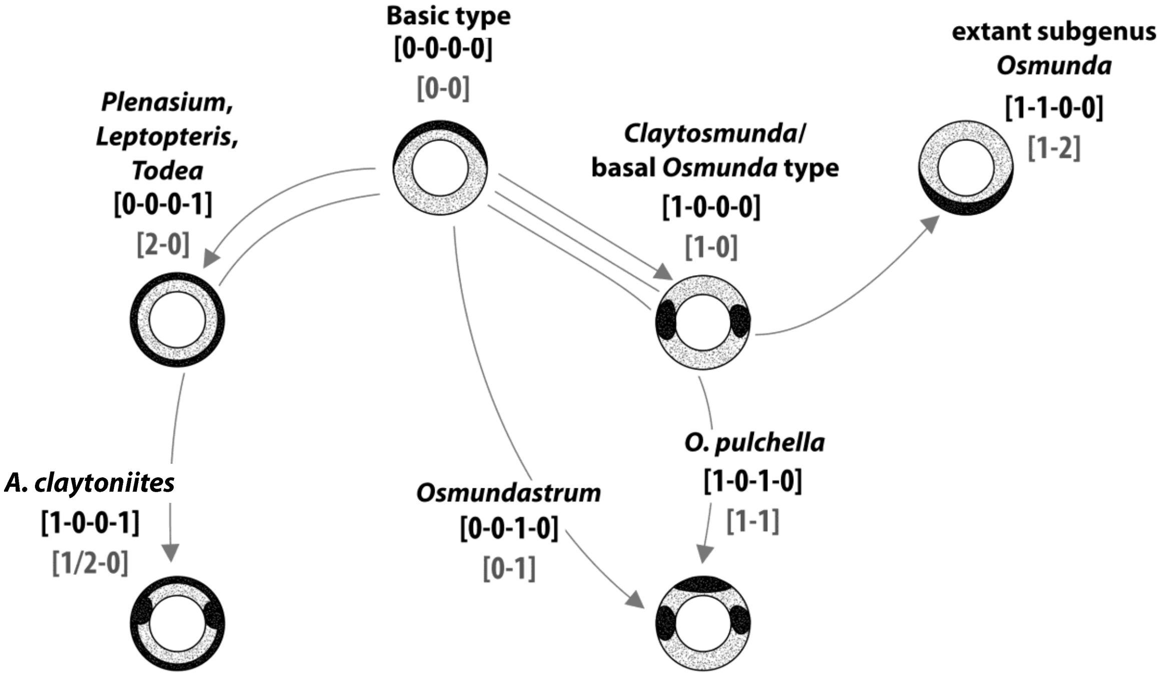

FIG. S1.1. Four-character binary coding (characters 26–29) and alternative 2-character ternary coding (in grey for comparison) describing the successive differentiation of patches of thick-walled fibres in petiole sclerenchyma rings of modern Osmundaceae. Arrows indicate the differentiation from a basal abaxial arch via intermediate configurations (in *O. pulchella*, *A. claytoniites*, and extant species of subgenus *Osmunda*) to the final configurations. Note that the coding allows distinction between the ultimately similar patterns in *O. pulchella* and members of subgenus *Osmundastrum.*

TAB. S1.4. Comparison of 4-character binary (above diagonal) and 2-character ternary coding schemes (below diagonal; see Fig. S1.1) of petiole sclerenchyma differentiation patterns.

	Basic type	*Plenasium, Leptopteris, Todea*	*A. claytoniites*	*Osmundastrum*	Ancestral *Osmunda/Claytosmunda*	Extant *Osmunda*	*O. pulchella*
Basic type	–	0.25	0.5	0.25	0.25	0.5	0.5
Plenasium, Leptopteris, Todea	0.5	–	0.25	0.5	0.5	0.75	0.75
A. claytoniites	0.5	0	–	0.75	0.5	0.5	0.5
Osmundastrum	0.5	1	1	–	0.5	0.5	0.25
Ancestral *Osmunda/Claytosmunda*	0.5	0.5	0	1	–	0.25	0.25
Extant *Osmunda*	1	1	0.5	1	0.5	–	0.5
O. pulchella	1	1	0.5	0.5	0.5	0.5	–

Character 27. — Two masses differentiating further into adaxial arch (modified coding of character II.K of Miller, 1971): (0) no; (1) yes.

See comment on character 25.

Character 28. — Abaxial arch or two masses differentiating into three masses (modified coding of character II.K of Miller, 1971): (no); (1) yes.

See comment on character 25.

Character 29. — Abaxial arch differentiating into ring (modified coding of character II.K of Miller, 1971; see ESA for details): (0) no; (1) yes.

See comment on character 25.

Character 30. — Sclerenchyma in the inner cortex of the petiole base (character II.l of Miller, 1971): (0) abundant; (1) absent.

Character 31 [excluded]. — Stipular expansions (character 20 of Wang *et al.*, 2013): (0) absent; (1) present .

Character 32. — Distinct sclerenchyma clusters in stipular expansions (character II.M of Miller, 1971): (0) absent; (1) oblong or irregular mass; (2) elongate strip.

Character 33. — Scattered sclerenchyma clusters in stipular expansions (character II.N of Miller, 1971): (0) absent; (1) present.

TAB. S1.5. Character coding for fossil and extant members of modern Osmundaceae in the morphological matrix.

Character	1	2	3	4	5	6	7	8	9	10	11	12	13	14	15	16	17	18	19	20	21	22	23	24	25	26	27	28	29	30	31	32	33
Leptopteris fraseri	0	2	0	1	?	0	0	2	0	0	0	1	0	0	1	1	0	0	1	0	1	0	0	0	1	0	0	0	1	1	1	0	1
Leptopteris hymenophylloides	0	2	0	1	?	0	0	2	0	0	0	1	0	0	1	1	0	0	1	1	1	0	0	0	1	0	0	0	1	1	1	1	1
Leptopteris wilkesiana	0	2	0	1	?	0	0	2	0	0	0	1	0	0	1	1	0	0	1	0	1	0	0	0	1	0	0	0	1	1	1	1	1
Leptopteris superba	0	2	0	1	?	0	0	2	0	0	0	1	0	0	1	1	0	0	1	1	1	0	0	0	1	0	0	0	1	1	1	1	1
Todea barbara	0	1	0	1	?	0	0	2	0	0	0	1	0	0	1	1	2	0	1	0	0	0	0	1	1	0	0	0	1	0	1	0	1
Todea papuana	0	1	0	1	?	?	?	?	?	?	?	?	?	?	?	?	?	?	?	?	?	?	?	?	1	?	?	?	?	0	1	0	1
Todea tidwellii	0	?	?	1	?	0	0	2	0	0	0	1	0	1	1	1	?	?	1	0	0	0	0	0	1	?	?	?	?	?	?	?	?
Osmunda cinnamomea	0	0	1/2	1	?	1	2	1	0	2	0	1	0	0	1	1	2	1	0	1	1	1	0	1	1	0	0	1	0	0/1	1	1	1
Osmunda cinnamomea (Neogene, USA)	0	?	?	1	?	0	0	1	0	1	0	1	0	0	1	1	2	0	0	0	1	1	0	1	1	0	0	1	0	0/1	1	1	1
Osmunda cinnamomea (Neogene, Japan)	0	?	?	1	?	0	?	0	0	0	0	1	0	?	1	1	2	0	0	0	1	1	0	1	1	0	0	1	0	1	1	1	1
Osmunda cinnamomea (Cretaceous, Canada)	0	?	?	1	?	0	0	0	0	2	0	1	0	0	1	1	2	1	0	1	1	1	0	1	1	0	0	1	0	0/1	1	1	1
Osmunda precinnamomea	0	?	?	1	?	0	0	0	0	1	0	1	0	0	1	1	2	1	0	1	1	1	0	1	1	0	0	1	0	0	1	1	1
Osmunda claytoniana	0	0	1	1	?	0	0	0	0	1	0	1	1	0	1	1	0	0	0	1	1	0	0	0	1	1	0	0	0	0	1	2	0
Ashicaulis (=*Millerocaulis* Vera) *claytoniites*	0	?	?	1	?	0	0	1	0	2	0	1	1	0	1	1	0	1	0	1	1	0	0	0	1	1	0	0	1	1	1	1	0
Osmunda banksiifolia	0	0	1	1	?	0	0	0	0	0	1	1	1	?	1	1	0	0	0	0	1	0	1	1	1	0	0	0	1	0	1	0	1
Osmunda bromeliifolia	0	0	1	1	?	0	0	0	0	2	1	1	1	?	1	1	0	0	0	0	1	0	1	1	1	0	0	0	1	0	1	1	1
Osmunda javanica	0	0	1	1	?	0	0	2	0	1	1	1	1	?	1	1	0	0	0	0	1	0	0	1	1	0	0	0	1	0	1	1	1
Osmunda vachellii	0	0	1	1	?	0	0	2	0	2	1	1	1	?	1	1	0	0	0	0	1	0	0	1	1	0	0	0	1	0	1	0	1
Osmunda arnoldii	0	?	?	1	?	0	0	2	0	0	1	1	1	?	1	1	0	0	0	0	1	0	1	1	1	0	0	0	1	0	1	0	1
Osmunda dowkeri	0	?	?	1	?	0	0	2	1	1	1	1	1	?	1	1	0	0	0	0	1	0	1	1	1	0	0	0	1	0	1	0	1
Osmunda japonica	0	1	1/2	1	?	0	0	0	0	0	0	1	1	1	1	1	0	0	0	0	1	1	0	0	1	1	1	0	0	1	1	2	1
Osmunda lancea	0	1	2	1	?	0	0	0	0	0	0	1	1	1	1	1	0	0	0	1	1	0	0	0	1	1	1	0	0	1	1	1	1
Osmunda regalis	0	1	1/2	1	?	0	0	1	0	0	0	1	1	1	1	1	0	0	0	1	1	1	0	0	1	1	1	0	0	0	1	2	0
Osmunda iliaensis	0	?	?	1	?	0	0	0	0	1	0	1	1	1	1	1	0	0	0	0	1	0	0	?	1	1	0	0	0	1	1	2	1
Osmunda nathorstii	0	?	?	?	?	?	?	?	?	?	0	?	?	?	?	?	?	?	?	?	1	1	?	?	1	1	0	0	0	0	1	2	0
Osmunda oregonensis	0	?	?	1	?	0	0	?	0	2	0	1	1	1	1	1	0	0	0	0	1	0	0	0	1	1	0	0	0	0	1	2	0
Osmunda pluma	0	?	?	1	?	0	0	?	0	0	0	1	1	1	1	1	0	0	0	1	1	0	0	0	1	1	0	0	0	0	1	2	1
Osmunda shimokawaensis	0	?	?	1	?	0	0	0	0	1	0	1	?	1	1	1	0	0	0	0	1	1	0	0	1	0	0	0	0	1	1	2	1
Osmunda wehrii	0	?	?	1	?	0	0	1	0	1	0	1	1	0	1	1	0	0	0	0	1	0	0	0	1	1	0	0	0	0	1	2	1
Osmunda pulchella	0	?	?	1	?	0	0	0	0	2	0	1	0	0	1	1	0	1	0	1	1	0	0	0	1	1	0	1	0	1	1	2	?
Osmunda kidstoni	0	?	?	?	?	?	?	?	?	?	?	?	?	?	?	?	?	?	?	?	1	1	?	0	?	?	?	?	?	1	1	1	1
Millerocaulis johnstonii	0	?	?	1	?	0	0	0	0	0	0	1	1	0	1	1	0	1	0	0	1	0	0	0	1	0	0	0	0	1	1	1	0
Millerocaulis liaoningensis	0	?	?	1	?	0	0	1	0	0	0	1	1	0/1	1	1	1	1	0	0	1	0	0	0	1	0	0	0	0	1	1	1	0
Ashicaulis (=*Millerocaulis* Vera) *wangii*	0	?	?	1	?	0	0	1	0	0	0	?	?	0	1	1	0	0	0	0	1	?	0	0	1	1	0	0	0	0	1	1	0
Ashicaulis (=*Millerocaulis* Vera) *plumites*	0	?	?	1	?	0	0	0	0	1	0	1	?	0	1	1	0	1	0	1	1	0	0	0	1	1	0	0	0	1	1	1	0

Set-up for phylogenetic analyses

Establishing support for alternative phylogenetic scenarios. — Support for (alternative) edges (branches) was established using nonparametric bootstrapping (BS; Felsenstein, 1985) under Least-squares (LS), Maximum parsimony (MP) and Maximum likelihood (ML) optimality criteria and posterior probabilities estimated via Bayesina inference (BI).

BS analysis under LS relied on 10,000 replicate trees inferred via the BioNJ algorithm (Gascuel, 1997) based on a matrix of mean (Hamming) pairwise morphological distances computed from the matrix using PAUP* (Swofford, 2002).

For MP-BS analysis, 10,000 replicate trees were inferred using PAUP* with the following settings (Müller, 2005). A single MP tree was inferred on each replicate matrix (option “MulTrees” deactivated) using heuristic search algorithm with option “AddSeq” set to “Furthest”. All other settings left to PAUP* defaults.

ML-BS support was estimated via the fast bootstrapping (option -x) implementation in RAxML v. 7.4.2 (Stamatakis, 2006b, Stamatakis *et al.*, 2008) and 10,000 replicates. Both available transition models for categorical (multistate) data, the general time-reversible model (Rodriguez *et al.*, 1990; -K GTR) and Lewis’ (2001) model (-K MK), were applied. The GTR model will allow for different transitions rates between character states, whereas the Lewis’ model estimate a single parameter, a general probability of character state change. For both models, we allowed for site-specific rate variation modelled via a Gamma (+Γ) distribution with 25 distinct rate categories (default in RAxML; -m MULTIGAMMA).

Bayesian analysis relied on MrBayes v. 3.2.2 (Ronquist and Huelsenbeck, 2003). 1,000,000 generations were computed in ten parallel runs with one (cold) Monte-Carlo Markov chain each and allowing for parameter and topology swapping between runs following the recommendations in the manual for analysis with few taxa and characters. The topology of every 1000th generation was sampled. Posterior probabilities take into account all sampled topologies from all ten runs saved for the first one, in total 10,000 sampled topologies. In contrast to molecular data sets, morphological data sets like the one used here converge directly to a plateau, why additional heated chains or high burn-in fractions are not needed (see folder Inferences/BI/OverviewRuns.xlsx).

Visualization of competing support patterns. — SplitsTree 4 (Huson and Bryant, 2006) was used to compute bipartition networks (Grimm *et al.*, 2006; option COUNT) based on the 10,000 LS-, ML- and MP-BS replicate trees and (input and output files can be found in folder Inferences/BipartionNetworks). In a bipartition network, a special form of consensus network

(Holland *et al.*, 2004), the edge length is proportional to the frequency of the corresponding split in the BS or Bayesian tree sample. Due to memory constraints and for simplicity, only such splits were considered which occurred in at least 20% of the BS replicate trees or Bayesian sampled topologies.

Set-up for re-analysis of the Metzgar et al. (2008) molecular data set. — Trees and bootstrap support were inferred with RAxML under ML using the concatenated data, each gene partition separately, and matrices in which one partition was deleted. RAxML was set to perform fast bootstrapping and initial tree inference under the per-site rates model, an approximation for the GTR + Γ model (Stamatakis, 2006a), with only the final model parameter optimization under GTR + Γ (-f a -m GTRCAT -x). Number of necessary BS replicates was determined using the extended majority-rule bootstop criterion (Pattengale *et al.*, 2009) implemented in RAxML (-# autoMRE) for each run. Model parameters were optimized individually for each gene partition and codon sites in the case of coding gene regions (in total 11 partitions used for the concatenated data set; see file “part” in folder Inferences/ML/Metzgar for details). Single-gene analyses and analysis of the concatenated data were run with our without outgroup (files labelled “…noOG…”) taxa. Bipartition networks (see last paragraph) were used to identify competing topologies and tabulate BS support for branches of interest.

Set-up for evolutionary placement algorithm

Molecular data. — The evolutionary placement algorithm (EPA) implemented in RAxML (Berger and Stamatakis, 2010, Berger *et al.*, 2011) was used to (1) investigate the position of the outgroup-inferred root based on the concatenated and single-gene molecular data sets and (2) to place the fossil taxa individually within the molecular framework of modern taxa using a probabilistic approach.

Root test. — The ingroup-only topology was used as reference tree and then the EPA was invoked to find the optimal placement of the outgroup sequences within the ingroup topology (-f v). Results are shown in main text, Figure 9.

Independent optimization of the placement of fossils within the molecular framework. — EPA was invoked using three different weighting schemes. ML-based weights for each character were estimated by RAxML using 1000 replicates (-f u -# 1000) under the GTR+Γ and MK+Γ transition models using a molecular-based species-consensus ML tree as reference (files

labelled …Moles… in folder Inferences). The use of species-consensus sequences was necessary since the molecular matrix of Metzgar *et al.* included several accessions for the same species exhibiting some intra-species variation. For one relatively recently described extant taxon, *Todea papuana*, the original description (Hennipman 1968) lacks information on the rhizome anatomy, hence, the taxon was excluded for EPA inferences (files labelled …MolesRed…). In addition to ML-defined weights, we also estimated MP-derived weights for completeness (-f U -# 1000). The option has been deactivated in newer versions of RAxML. MP weights of individual characters can easily collapse due to homoplasy inherent to morphological data sets (G. Grimm, pers. observ.) and have thus been judged to be inferior to ML weights and dropped by the inventors of the EPA (A. Stamatakis, pers. comm., 2014; weighting scheme not included in Berger and Stamatakis, 2010).

Additional literature cited

Berger SA, Krompass D, Stamatakis A. 2011. Performance, accuracy, and web server for evolutionary placement of short sequence reads under Maximum Likelihood. *Systematic Biology* **60**: 291–302.

Berger SA, Stamatakis A. 2010. Accuracy of morphology-based phylogenetic fossil placement under Maximum Likelihood. *IEEE/ACS International Conference on Computer Systems and Applications (AICCSA)* IEEE.

de Hoon MJL, Imoto S, Nolan J, Miyano S. 2004. Open source clustering software. *Bioinformatics* **20:** 1453–1454.

Gascuel O. 1997. BIONJ: An improved version of the NJ algorithm based on a simple model of sequence data. *Molecular Biology and Evolution* **14**: 685–695.

Grimm GW, Renner SS, Stamatakis A, Hemleben V. 2006. A nuclear ribosomal DNA phylogeny of *Acer* inferred with maximum likelihood, splits graphs, and motif analyses of 606 sequences. *Evolutionary Bioinformatics* **2**: 279–294.

Hennipman E. 1968. A new *Todea* from New Guinea, with remarks on the generic delimitation in recent Osmundaceae (Filices). *Blumea* **16**: 105–108.

Holland B, Huber KT, Moulton V, Lockhart PJ. 2004. Using consensus networks to visualize contradictory evidence for species phylogeny. *Molecular Biology and Evolution* **21**: 1459–1461.

Huson DH, Bryant D. 2006. Application of phylogenetic networks in evolutionary studies. *Molecular Biology and Evolution* **23**: 254–267.

Lewis PO. 2001. A likelihood approach to estimating phylogeny from discrete morphological character data. *Systematic Biology* **50**: 913–925.

Metzgar JS, Skog JE, Zimmer EA, Pryer KM. 2008. The paraphyly of *Osmunda* is confirmed by phylogenetic analyses of seven plastid loci. *Systematic Botany* **33:** 31–36.

Müller KF. 2005. The efficiency of different search strategies for estimating parsimony, jackknife, bootstrap, and Bremer support. *BMC Evolutionary Biology* **5**: 58.

Pattengale ND, Masoud A, Bininda-Emonds ORP, Moret BME, Stamatakis A. 2009. How many bootstrap replicates are necessary? In: Batzoglou S, ed. *RECOMB 2009.* Berlin, Heidelberg: Springer–Verlag.

Rodriguez F, Oliver JL, Marin A, Medina JR. 1990. The general stochastic model of nucleotide substitution. *Journal of Theoretical Biology* **142**: 485–501.

Ronquist F, Huelsenbeck JP. 2003. MrBayes 3: Bayesian phylogenetic inference under mixed models. *Bioinformatics* **19**: 1572–1574.

Stamatakis A. 2006a. Phylogenetic models of rate heterogeneity: A high performance computing perspective. *Proceedings of 20th IEEE/ACM International Parallel and Distributed Processing Symposium (IPDPS2006), High Performance Computational Biology Workshop.* Rhodos, Greece, April 2006.

Stamatakis A. 2006b. RAxML-VI-HPC: Maximum-Likelihood-based phylogenetic analyses with thousands of taxa and mixed models. *Bioinformatics* **22**: 2688–2690.

Stamatakis A, Hoover P, Rougemont J. 2008. A rapid bootstrap algorithm for the RAxML web servers. *Systematic Biology* **57**: 758–771.

Swofford DL. 2002. PAUP*: Phylogenetic Analysis Using Parsimony (and Other Methods) 4.0 Beta. Sunderland, MA: Sinauer Associates.
