# Supplementary material for: Osmunda pulchella sp. nov. from the Jurassic of Sweden—reconciling molecular and fossil evidence in the phylogeny of modern royal ferns (Osmundaceae)
Source: BMC Evol Biol. 2015 Jun 30;15:126. doi: 10.1186/s12862-015-0400-7 (PMC4487210; doi:10.1186/s12862-015-0400-7)
Supplement: Additional file 2: — Neighbour net inferred from uncorrected pairwise distances based on the concatenated data set of Metzgar et al. (2008). Bootstrap support of (alternative) splits is annotated. Note the occurrence of four genetically distinct lineages; the splits that place O. cinnamomea as sister to all other Osmundaceae received strong support only from atpA and rbcL partitions. [file 12862_2015_400_MOESM2_ESM.pdf]

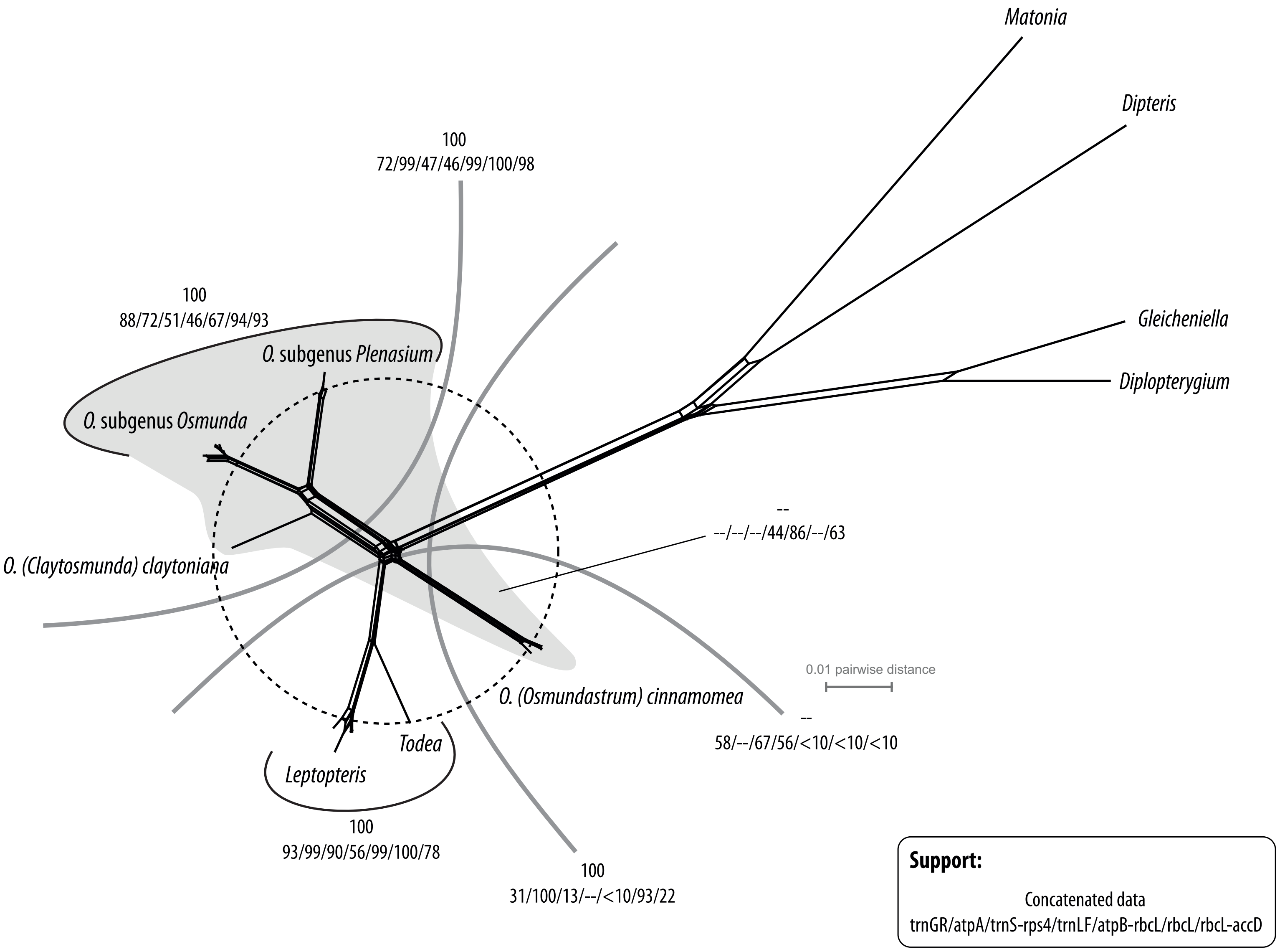

Bomfleur *et al.*, Fig. S1. Neighbour net inferred from uncorrected pairwise distances based on the concatenated data set of Metzgar *et al.* (2008). Bootstrap support of (alternative) splits is annotated. Note the occurrence of four genetically distinct lineages; the splits that places *O. cinnamomea* as sister to all other Osmundaceae only received high support from *atpA* and *rbcL* partitions.
